# Intrinsic lipolysis rate for systematic design of lipid-based formulations

**DOI:** 10.1007/s13346-022-01246-y

**Published:** 2022-10-08

**Authors:** Ann-Christin Jacobsen, Aleksei Kabedev, Patrick D. Sinko, Johan E. Palm, Christel A. S. Bergström, Alexandra Teleki

**Affiliations:** 1 The Swedish Drug Delivery Center, Department of Pharmacy, Uppsala University, Uppsala, Sweden; 2 Department of Pharmacy, Uppsala University, Uppsala, Sweden; 3 Pharmaceutical Technology & Development, Oral Product Development, Gothenburg, Operations, AstraZeneca Sweden; 4 Department of Pharmacy, Science for Life Laboratory, Uppsala University, Box 580, 75123 Uppsala, Sweden

**Keywords:** Lipid digestion, Nanoemulsion, Lipid-based formulations, Molecular dynamics simulations, Lipolysis, Drug development

## Abstract

Lipid-based formulations (LBFs) are used by the pharmaceutical industry in oral delivery systems for both poorly water-soluble drugs and biologics. Digestibility is key for the performance of LBFs and in vitro lipolysis is commonly used to compare the digestibility of LBFs. Results from in vitro lipolysis experiments depend highly on the experimental conditions and formulation characteristics, such as droplet size (which defines the surface area available for digestion) and interfacial structure. This study introduced the intrinsic lipolysis rate (ILR) as a surface area-independent approach to compare lipid digestibility. Pure acylglycerol nanoemulsions, stabilized with polysorbate 80 at low concentration, were formulated and digested according to a standardized pH–stat lipolysis protocol. A methodology originally developed to calculate the intrinsic dissolution rate of poorly water-soluble drugs was adapted for the rapid calculation of ILR from lipolysis data. The impact of surfactant concentration on the apparent lipolysis rate and lipid structure on ILR was systematically investigated. The surfactant polysorbate 80 inhibited lipolysis of tricaprylin nanoemulsions in a concentration-dependent manner. Coarse-grained molecular dynamics simulations supported these experimental observations. In the absence of bile and phospholipids, tricaprylin was shielded from lipase at 0.25% polysorbate 80. In contrast, the inclusion of bile salt and phospholipid increased the surfactant-free area and improved the colloidal presentation of the lipids to the enzyme, especially at 0.125% polysorbate 80. At a constant and low surfactant content, acylglycerol digestibility increased with decreasing acyl chain length, decreased esterification, and increasing unsaturation. The calculated ILR of pure acylglycerols was successfully used to accurately predict the IRL of binary lipid mixtures. The ILR measurements hold great promise as an efficient method supporting pharmaceutical formulation scientists in the design of LBFs with specific digestion profiles.

**Graphical abstract:**

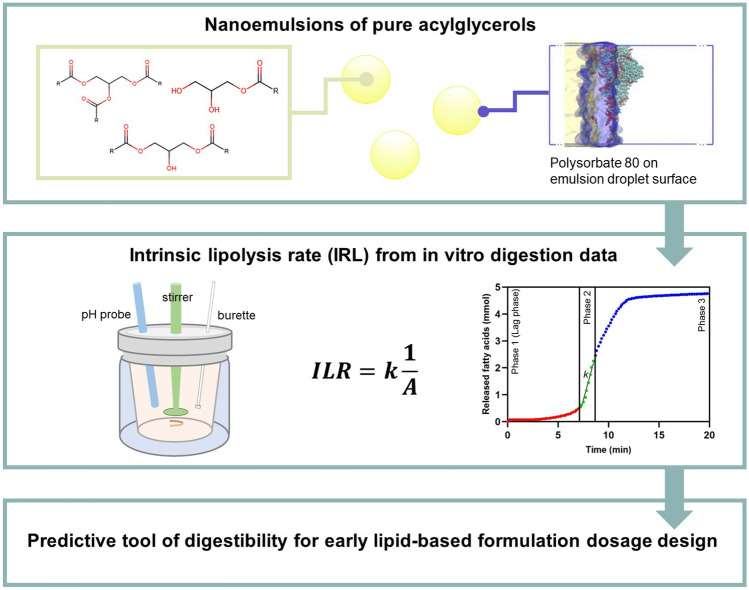

**Supplementary Information:**

The online version contains supplementary material available at 10.1007/s13346-022-01246-y.

## Introduction

Lipid excipients such as acylglycerols, phospholipids, and surfactants are used to prepare bioenabling pharmaceutical formulations of highly variable complexity, here collectively termed lipid-based formulations (LBFs). Lipid solutions containing one drug and one lipid as the solvent can be considered the simplest LBF [1]. In contrast, self-emulsifying drug delivery systems [2] and solid lipid nanoparticles [3] typically contain several constituents and require more intricate preparation methods. Within oral delivery, LBFs are traditionally used for poorly water-soluble small molecule drugs. Dissolving drugs with low aqueous solubility in a lipid phase can enhance oral absorption by circumventing the gastrointestinal dissolution step [4]. More recently, LBFs have been explored as delivery systems for drugs with low lipid solubility, such as high melting-point drugs (i.e., “brick dust” molecules) [5] and biologics [6]. In 2020, the US Food and Drug Administration approved Mycapssa, an enteric coated capsule containing an oily suspension of octreotide, a cyclic octapeptide, for the treatment of acromegaly [7].

Oral administration of LBFs triggers physiological responses related to the digestion and absorption of lipids, which in turn influences formulation performance [8]. Important physiological responses include the secretion of bile and pancreatic juice into the intestinal lumen. Bile contains bile salts, phospholipids and cholesterol, all acting as emulsifiers for lipids. Enzymes in pancreatic juice, such as pancreatic lipase, break the ester bonds of lipids (i.e., triacylglycerols) at the lipid-water interface [9]. Specifically, the pancreatic lipase-colipase complex cleaves the ester bonds of triacylglycerols at the sn-1 and sn-3 positions, releasing two fatty acids and one 2-monoacylglycerol [10]. Other lipases in pancreatic juice, such as pancreatic lipase-related protein 2 (PLRP2) and carboxyl ester hydrolase (CEH), are active towards partial acylglycerols (i.e., mono and diacylglycerols) and PEG esters [11]. Partial acylglycerols and PEG esters are common constituents of many LBFs.

The rate and extent of lipid digestion, i.e., lipolysis, is an important parameter influencing the performance of LBFs. The digestion of lipids into fatty acids and other lipolysis products affects the solubilization capacity of the intestinal fluid. This can be decisive for formulation performance [12, 13]. The solubilization capacity of the intestinal fluid may decrease or increase depending on the properties of the drug. Decreased solubilization capacity can lead to drug precipitation which is detrimental for formulation performance. Furthermore, lipid digestion can enhance intestinal permeability, which affects oral absorption of poorly permeable drugs (e.g., biologics including peptides). This increased intestinal permeability can be an indirect result of a decreased expression of tight junction proteins [14] or release of cholecystokinin [15, 16]. It might also result directly from the permeation-enhancing effect of digestion products such as fatty acids (i.e., multimodal permeation enhancement mechanism acting both on trans- and paracellular transport) and monoacylglycerols (i.e., transcellular permeation enhancer) [17]. For example, the lipase mediated digestion of triacylglycerols significantly affects the oral absorption of cefoxitin in rats [18]. Digestion of tricaprylin and monocaprylin (medium chain acylglycerols) in the commercial Mycapssa formulation [7] could contribute to the oral absorption of octreotide, which is mediated by permeation enhancement.

Since lipolysis is key for the performance of LBFs, in vitro lipolysis methods have been introduced for formulation screening and optimization [13]. The experimental conditions (e.g., the concentration of bile salt, Ca^2+^ and lipase, as well as source and type of lipase) strongly influence the outcome of in vitro lipolysis. This has led to the development of standardized protocols enabling inter-laboratory comparison. One example is the pH–stat method established by the Lipid Formulation Classification System (LFCS) Consortium. In this method, lipolysis, which causes a drop in pH, is determined via titration with dilute sodium hydroxide [19]. Another protocol accounts for differences in experimental conditions by comparing the digestion of lipid excipients to a standard lipid (i.e., Miglyol 812, a medium-chain triglyceride). The resulting “relative lipolysis half-life” should be independent of the experimental conditions used [20]. Still, direct comparison of the digestibility of lipid excipients and LBFs remains challenging. Lipolysis is an interfacial reaction [9] governed by the lipid droplet size [21, 22], because the size determines the total surface area available for digestion. In turn, the lipid droplet or lipid particle size depends on formulation composition, preparation method, and dispersion of the LBF in the lipolysis medium (i.e., stirrer type and stirring rate) [23]. Preparation methods such as homogenization (e.g., high-pressure homogenization and ultrasonication) offer precise control over droplet size. On the other hand, formulation composition is the decisive factor for droplet size of lipid solutions that self-disperse in the intestinal fluid. A high content of hydrophilic or amphiphilic components (i.e., surfactants) offers enhanced control over droplet size, as described in the LFCS [4]. However, surfactants at the lipid-water interface can inhibit lipolysis, complicating comparison of the digestibility of LBFs [24–27]. Additionally, it should be considered that most LBF excipients are mixtures of different lipids (e.g. medium chain mono-, di- and triglycerides). This further complicates the characterization and understanding of the digestion of lipid excipients and LBFs. For example, Capmul MCM in Mycapssa is a mixture of mono-, di- and triacylglycerols of caprylic and capric acid.

Mathematical modeling of in vitro lipid digestion kinetics has been investigated both in the field of food and pharmaceutical sciences [20, 28–30]. Some of the earlier investigations of lipid digestion kinetics use Michaelis–Menten kinetics to model lipid hydrolysis (e.g., [28]). First or pseudo-first order reaction models are the simplest and most widely used to model experimental lipolysis data [30]. The pseudo first order kinetic model described by Li and McClements, and later corrected by Gaucel et al. assumes a constant number of lipid droplets of equal size to model the free fatty acid release over time in the experimental pH–stat lipolysis method [31, 32]. The model by Giang et al. accounts for the effect of oil droplet coalescence on lipolysis kinetics [33]. Lipid digestion is an interfacial process, and Jurado et al. expanded the scope of lipid digestion models to include interfacial kinetics as exemplified for tributyrin [34]. Other models, like the mechanism-based multi-response models, capture the interaction of the multiple digestion species formed during in vitro lipolysis more accurately than the early first-order reaction models [35]. However, the validation of these multi-response models requires more advanced analytics to experimentally quantify multiple lipid digestion species. Overall, these models of in vitro lipid digestion have not been widely adopted by pharmaceutical scientists in the development and understanding of LBFs. Thus, there is a need for simple, yet accurate, approaches that can guide LBF development. These approaches need to focus on lipid excipients commonly used in oral drug delivery systems, rather than lipids commonly found in complex foods. This would aid the development of LBFs of the large fraction of poorly soluble and poorly permeable compounds in the pipeline, whereby oral absorption of those compounds hopefully can be enhanced.

In this study, we introduce the intrinsic lipolysis rate (ILR) to enable direct comparison of the digestibility of lipids and to support systematic design of LBFs. A methodology developed for the suspension-based intrinsic dissolution rate [36, 37] was adapted here for lipid digestion. Nanoemulsions of controlled lipid droplet size were prepared with pure acylglycerols that differed in acyl chain length, esterification, and unsaturation. The lipid-water interface in these nanoemulsions was stabilized by low concentrations of the non-ionic surfactant polysorbate 80. The influence of the surfactant on lipid digestibility was studied both experimentally and computationally to elucidate its impact on interfacial reactions. Lipolysis of the nanoemulsions was studied by the LFCS pH–stat method [19] and the total surface area available for lipid digestion was estimated from the droplet size. From these data, a surface area-independent lipolysis rate, the ILR, was derived by adapting the suspension-based intrinsic dissolution rate methodology. To the best of our knowledge, our study contains the largest collection of lipolysis data of pure acylglycerols to date. We demonstrate that the ILR can be used to predict the digestibility of lipid mixtures from this data set. Thus, the ILR may aid formulation scientists to design multicomponent LBFs with known digestibility.

## Materials and methods

### Acylglycerols and lipolysis medium

Acylglycerols of at least 95% purity were used in this study. Tricaprylin (purity > 99%) was purchased from Sigma Aldrich (St. Louis, MO, USA). 1-monocaprylin (> 98%), 1-monocaprin (> 98%), 1-monolaurin (> 98%), tricaprin (> 98%), 1,3-dilaurin ( > 96%), trilaurin (> 98%), and trilinolein (> 95%) were obtained from TCI Europe (Zwijndrecht, Belgium). Triolein (> 95%) was purchased from Fisher Scientific (Waltham, MA, USA). The structure and properties of the acylglycerols are given in Table S1. A buffer consisting of 2 mM Tris-maleate, 1.4 mM CaCl_2_ and 150 mM NaCl with a pH of 6.5 was used in this study and is referred to as “lipolysis buffer.” All buffer components were purchased from Sigma Aldrich (St. Louis, MO, USA) and were of analytical grade. The medium used for in vitro lipolysis consisted of lipolysis buffer supplemented with fasted state simulated intestinal fluid (FaSSIF) instant powder (biorelevant.com, London, UK); FaSSIF instant powder comprises the bile salt sodium taurocholate and lecithin. The concentration of sodium taurocholate and phospholipid in the lipolysis medium was 6 and 1.5 mM, respectively.

### Preparation of acylglycerol nanoemulsions

Acylglycerol nanoemulsions (droplet sizes 200–400 nm) were prepared by ultrasonication. The emulsions contained a single acylglycerol or a mixture of two acylglycerols and were stabilized with the non-ionic surfactant polysorbate 80 (Sigma Aldrich, St. Louis, MO, USA). The composition of all nanoemulsions was selected to yield 5 mmol fatty acids upon complete digestion. It was assumed that triacylglycerols and 1,3-diacylglycerols release two fatty acids and 1-monoacylglycerols release one fatty acid per molecule. To prepare the nanoemulsions, the acylglycerol(s) were weighed into a beaker, a solution of polysorbate 80 in lipolysis buffer was added, and the beaker covered with parafilm. The mixture was sonicated in a temperature-controlled sonication bath, for 15 min at a temperature 10 °C above the melting point of the acylglycerol(s) (Table S1). The mixture was then immediately ultrasonicated using an ultrasonic liquid processor (Vibra Cell™, Sonics, Newtown, CT, USA) equipped with a Ø12-mm tip probe. Ultrasonication was conducted for 0.5–5 min at 50% amplitude in pulse mode (5 s on/20 s off) until no further reduction in droplet size was detected (Table S2). Three sets of experiments were conducted with the acylglycerols; the nanoemulsion compositions are given in Table 1. Nanoemulsions containing 125 mM tricaprylin and 0.25–4% (w/w) polysorbate 80 were prepared to study the influence of polysorbate 80 concentration on digestion (data set 1). To study the influence of lipid properties on digestion, nanoemulsions containing 125 mM of a single 1,3-diacylglycerol or triacylglycerol and 0.5% (w/w) polysorbate 80 were prepared (data set 2). Binary nanoemulsions of acylglycerols were prepared to explore the predictive potential of the ILR (data set 3). Binary nanoemulsions of 1,3-diacylglycerol and triacylglycerol contained 125 mM acylglycerol and 0.5% (w/w) polysorbate 80. Binary nanoemulsions of 1-monoacylglycerols and triacylglycerol contained 131.25 or 137.5 mM acylglycerol and 0.5% (w/w) polysorbate 80.

**Table 1:** Composition, droplet size, and digestion rate constants of acylglycerol nanoemulsions. The hydrodynamic diameter and the droplet polydispersity index (PDI) were measured by DLS. The digestion rate constants were determined using the ILR methodology (Eqs. 1–6) or the first-order mechanistic model proposed by Li and McClements and corrected by Gaucel et al. (Eq. 9)

	**Acylglycerol(s) in nanoemulsion**	**Lipid concentration** **(mM)**	**Polysorbate 80 concentration** **(%; w/w)**	**Hydrodynamic** **diameter** **(nm)**	**PDI**	**1**^**st**^** order digestion rate constant (ILR)**^**a**^ **(× 10**^**–3**^** µmol min**^**−1**^** cm**^**−2**^**)**	**1**^**st**^** order digestion rate constant**^**b**^ **(× 10**^**–3**^** µmol min**^**−1**^** cm**^**−2**^**)**
**1. Effect of polysorbate 80 concentration**	Tricaprylin (C8:0)	125	0.125	304 ± 10	0.23 ± 0.01	8.8 ± 0.1	N/A
			0.25	250 ± 7	0.26 ± 0.006	6.3 ± 0.3	
			0.5	233 ± 3	0.24 ± 0.007	5.2 ± 0.4	
			1	211 ± 4	0.23 ± 0.004	3.6 ± 0.3	
			1.5	204 ± 5	0.22 ± 0.009	2.9 ± 0.3	
			2	207 ± 2	0.22 ± 0.016	2.6 ± 0.4	
**2. Pure acylglycerols**	Tricaprylin (C8:0)	125	0.25	250 ± 7	0.26 ± 0.006	6.3 ± 0.3	4.1 ± 0.3
	Tricaprin (C10:0)			281 ± 4	0.26 ± 0.002	2.8 ± 0.4	1.8 ± 0.3
	1,3 dilaurin (C12:0)			280 ± 18	0.25 ± 0.016	2.9 ± 0.3	1.8 ± 0.6
	Trilaurin (C12:0)			298 ± 12	0.24 ± 0.009	0.9 ± 0.09	1.4 ± 0.2
	Triolein (C18:1)			400 ± 10	0.26 ± 0.01	0.26 ± 0.03	0.35 ± 0.04
	Trilinolein (C18:2)			363 ± 13	0.23 ± 0.009	0.44 ± 0.04	0.59 ± 0.02
**3. Binary nanoemulsions**	Tricaprylin (C8:0), tricaprin (C10:0)	62.5	0.25	255 ± 3	0.23 ± 0.01	4.4 ± 0.2	3.6 ± 0.1
		62.5					
	Tricaprylin (C8:0), tricaprin (C10:0)	31.25		295 ± 26	0.26 ± 0.014	3.5 ± 0.8	2.2 ± 0.4
		93.75					
	Tricaprylin (C8:0), triolein (C18:1)	62.5		316 ± 13	0.23 ± 0.019	2.8 ± 0.1	1.6 ± 0.2
		62.5					
	1,3 dilaurin (C12:0), trilaurin (C12:0)	62.5		282 ± 8	0.23 ± 0.014	2.1 ± 0.2	2.6 ± 0.03
		62.5					
	Trilaurin (C12:0), 1-monocaprylin (C8:0)	112.5		216 ± 8	0.18 ± 0.036	2.3 ± 0.2	N/A^c^
		25					
	Trilaurin (C12:0), 1-monocaprin (C10:0)	118.75		234 ± 5	0.19 ± 0.035	1.4 ± 0.07	
		12.5					
	Trilaurin (C12:0), 1-monolaurin (C12:0)	118.75		236 ± 2	0.23 ± 0.005	1.3 ± 0.1	
		12.5					
	Triolein (C18:1), 1-monocaprin (C10:0)	118.5		299 ± 6	0.17 ± 0.033	0.7 ± 0.07	
		12.5					

^a^Calculated using the IRL methodology, n = 3, mean ± standard deviation

^b^Calculated using an iterative least squares multiple linear regression fitting algorithm in MATLAB 2019a. The model equation was the model proposed by Li and McClements and corrected by Gaucel et al. (see Eq. 9) [31, 32], *n* = 3, mean ± standard deviation

^c^The model by Li and McClements assumes that two fatty acids are released per triacylglycerol. This is not applicable for 1-monoacylglycerols

### In vitro lipolysis of acylglycerol nanoemulsions

Freshly prepared acylglycerol nanoemulsions were digested in vitro by pH–stat lipolysis [19]. For this, a titrator (907 Titrando, Metrohm, Herisau, Switzerland) connected to a 10-ml burette, a pH electrode (iUnitrode with Pt 1000, Metrohm, Herisau, Switzerland) and a propeller stirrer was used. Nanoemulsion (20 mL), corresponding to a theoretical total fatty acid release of 5 mmol, and 20 ml lipolysis medium were added to a conical titration vessel (6.1418.220, Methrohm, Herisau, Switzerland) connected to a water bath set to 37 °C. The final concentration of phospholipid and sodium taurocholate in the lipolysis vessel containing the nanoemulsion and the lipolysis medium was 0.75 and 3 mM, respectively.

Prior to lipolysis, the mixture of the nanoemulsion and lipolysis medium was stirred for 10 min. During this time, the pH was manually adjusted to 6.5 with 0.1 M NaOH and a 10 µL sample was taken to determine the size of the acylglycerol nanoemulsion droplets. The sample was diluted 1:100 with lipolysis buffer and the hydrodynamic diameter of the nanoemulsion droplets was measured by dynamic light scattering (DLS) using a Litesizer 500 (Anton Paar, Graz, Austria). DLS measurements were conducted at 37 °C using the instrument’s automatic setting for adjustment of the focus and measurement angle. In vitro lipolysis was initiated by addition of 4.5 ml pancreatic extract. The pancreatic extract was prepared by suspending 1.2 g pancreatin from porcine pancreas (8 × USP specifications, Sigma Aldrich, St. Louis, MO, USA) in 6 ml of lipolysis buffer. The suspension was centrifuged for 15 min at 2465 g and 5 °C. Thereafter, the release of ionized fatty acids was followed over 90 min via auto-titration using 0.6 M NaOH as the titrant. After lipolysis, the titrator was set to raise the pH to 9 for estimation of the fraction of unionized fatty acids. To correct for the fatty acids released from the digestion of phospholipids, the lipolysis medium was digested in the absence of acylglycerols. After 1:1 dilution with lipolysis buffer, the lipolysis medium was digested following the same procedure except that 0.2 M NaOH was used as the titrant. All experiments were carried out in triplicate. All data, including hydrodynamic diameters from DLS measurement and lipolysis data, are presented as mean ± standard deviation. Unpaired student’s t-test was used to identify statistically significant differences between two groups, where p < 0.05 was considered significant.

### Calculation of the intrinsic lipolysis rate

The intrinsic lipolysis rate (ILR; µmol min^−1^ cm^−2^) was calculated analogous to the methodology developed for the suspension-based dissolution rate [36]:

1 $$ILR=k\frac1A$$

where, *k* (µmol min^−1^) is the initial slope of the lipolysis curve at the point where the fatty acid release is at its highest rate, and *A* (cm^2^) is the total lipid droplet surface area available for lipolysis. The lipolysis curve refers to the cumulative amount of fatty acid released over time including ionized and unionized fatty acids and corrected for the fatty acids released from the digestion of phospholipids. Here, it is assumed that the ratio between ionized and unionized fatty acids does not change during the experiment. Hence, the ratio between ionized fatty acids (determined by direct titration) and unionized fatty acids (determined by raising the pH to 9) at the end of the experiment can be used to estimate the total amount of fatty acids released over time. The total droplet surface area available for lipolysis (*A*) was calculated from the surface area of a single droplet (SA_droplet_) and the total number of droplets (n_droplets_):

2 $$A=n_{droplets}SA_{droplet}$$

The total number of droplets was taken as the ratio between the total volume of acylglycerol (V_acylglycerols_) and the volume of a single droplet (V_droplet_):

3 $$n_{droplets}=\frac{V_{acylglycerol}}{V_{droplet}}$$

4 $$V_{acylg lycerol}=\frac{m}{\rho}$$

5 $$V_{droplet}=\frac{4\pi r^3}{3}$$

In Eq. (4), *m* is the mass of acylglycerol (g) and ρ is the density of the acylglycerol (g cm^-3^; Table S2). The radius of a single droplet (*r*; cm) was obtained from the DLS measurements of the hydrodynamic diameter. The surface area of a single droplet was calculated using Eq. (6):

6 $$SA_{droplet}=4\pi r^2$$

### Predictions based on the intrinsic lipolysis rate

The potential of the ILR to predict the digestibility of LBFs was explored by investigating the lipolysis of nanoemulsions containing binary mixtures of acylglycerols (data set 3). As a basis for comparison, the ILR of binary acylglycerol emulsions was determined experimentally as described above. To calculate the total surface area available for digestion, it was assumed that the density of the acylglycerol mixture would reflect the density of the components and could be estimated from their molar ratio assuming ideal mixing (Table S2). For mixtures containing small amounts of 1-monoacylglycerol, the density of the triacylglycerol was used. A predicted ILR of the binary mixtures (*ILR*_*mix*_) was calculated from the ILR of the pure components (here denoted with subscripts *A* and *B*) based on the molar fractions of fatty acids (*x)*:

7 $$ILR_{mix}=x_{A}\cdot ILR_{A}+X_{B}\cdot ILR_{B}$$

The molar fractions of fatty acids (*x*) were calculated by dividing the theoretical maximum number of fatty acids released from a specific acylglycerol (*n*_*fatty acid acylglycerol*_; mmol) by the total theoretical maximum number of fatty acids released from both acylglycerols (*n*_*fatty acid total*_; in all experiments 5 mmol):

8 $$x=\frac{n_{fatty\;acid\;acylglycerol}}{n_{fatty\; acid\;total}}$$

Further, Eqs. (7) and (8) were used to predict the ILR of pure acylglycerols that did not form emulsions with well-defined droplets on their own (i.e., 1-monocaprylin, 1-monocaprin and 1-monolaurin). Thus, binary mixtures of a triacylglycerol and a 1-monoacylglycerol were prepared and the mixed ILR was determined experimentally as described above. As the ILR of one of the pure components and the molar fractions of fatty acids are known, the ILR of the second pure component can be predicted using Eq. (7).

### Mechanistic predictions of intrinsic lipolysis rate

To verify the ILR calculations, a digestion rate constant was predicted using an iterative multiple linear regression fitting algorithm (fitnlm, Statistics and Machine Learning Toolbox, MATLAB 2019a). A copy of the executable code is provided in supplementary material (Appendix S1) and more information on fitnlm can be found on the online MATLAB documentation. The equation was the model proposed by Li and Clements [31] and corrected by Gaucel and co-workers [32] as seen in Eq. (9),

9 $$\Phi=\Phi_{max}\left( {1-max}\left\{ {0,\left( {1-\frac{kM}{d_{0}\rho_{0}}}t \right)^3} \right\} \right)$$

where Φ is the fraction of fatty acids released, *k* is the first order rate constant (mol s^−1^ m^−2^), *M* is the molecular weight of the acylglycerol (kg mol^−1^), *d*_*0*_ is the initial emulsion droplet diameter, and *ρ*_0_ is the density of the acylglycerol (kg m^−3^). The ILR digestion rate constant has the units of a first order rate constant, and is thus comparable to the digestion rate constant derived from the Li and McClements model for digestion of lipids [31, 32]. Two parameters were fit against the fraction of fatty acids released-time course data, the maximum extent of digestion (Φ_*max*_) and the first order digestion rate constant (*k*), as described by Li and McClements [31, 32]. Initial values for the fit parameters were tested above and below the expected range of valid solutions and no significant differences in the predicted fit parameters was observed. All other parameters were taken from digital repositories (molecular weight/density, PubChem), or measured experimentally using DLS (initial droplet size). Graphical comparisons of all fittings can be found in the supplementary material (Figs. S1-S3).

### Molecular dynamics simulations

Computational simulations were used to study the surface coverage of lipid droplets with polysorbate 80 at various concentrations and to evaluate the impact of this surfactant on interfacial interactions with water and lipase molecules. Coarse-grained molecular dynamics (MD) simulations were performed with Gromacs 2018 [38] using the Martini 2.2 force field [39]. In that force field every group of four heavy atoms, or a group of four water molecules, is represented with a single bead. This approach lowers computational costs of the simulations. Tricaprylin was selected as the model acylglycerol in the MD simulations for comparison with the experimental data series with varying surfactant concentration (Table 1). Sodium taurocholate and 1,2-dilinoleoyl-sn-glycero-3-phosphatidylcholine (hereafter referred to as phosphatidylcholine) were used to represent the bile salt and phospholipid, respectively, present in the lipolysis buffer. Topologies of sodium taurocholate, phosphatidylcholine and tricaprylin molecules (Fig. S4, Table S4) were available from our previous studies [40, 41]. Polysorbate 80 topology (Fig. S4, Table S5) was adopted from models developed by other groups [42, 43].

The lipid droplets were represented by a stack of the lipids equal to a small fraction of the full-size droplet present in the lipolysis vessel to reduce computational time (Fig. S5). Tricaprylin phase was set up under xy-periodic boundary condition and solvated above and below in z-dimension. Surfactant was then added to the layer above the triacylglycerols in close proximity to the surface, so that its entire mass would be in contact with tricaprylin. Later, sodium taurocholate (3 mM) and phosphatidylcholine (0.75 mM) were added randomly to the entire water volume to reflect the composition of the lipolysis medium. The number of polysorbate 80 molecules was chosen with the assumption that all surfactant molecules would end up on the surface of the lipid colloids. It was thus calculated based on the size of the lipid droplet and the polysorbate 80 concentration (for information on the calculation, the reader is referred to Fig. S5 in the supplementary material). This represents the maximum possible surface coverage and might overestimate the real scenario in which pure polysorbate 80 micelles could co-exist with the mixed nanoemulsion droplets. Full partitioning of the surfactant likely overestimates the real portion of polysorbate 80 at the lipid droplet surface (the other extremum being no polysorbate 80 covering the lipid droplets). The partial coverage by polysorbate 80 molecules might be estimated from the simulations with increasing surfactant concentration (0.125, 0.25 and 0.5% polysorbate 80). All simulations were performed at 37 °C, with semi-isotropic pressure coupling, at 1 bar. Compressibility of 0 and 3e^−4^ bar^−1^ were applied in xy-plane and z-dimension, respectively. The former value is needed to prevent stretching of the lipid layer and absorption of an unrealistic number of external molecules. V-rescale thermostat [44] and Parrinello-Rahman barostat [45] were applied for the production simulations. After energy minimization and system equilibration, the production runs of 3 microseconds were performed to obtain the equilibrated adsorption of polysorbate 80 molecules, sodium taurocholate and phosphatidylcholine. A periodic boundary condition in all three box directions was applied in the simulations. Average simulation box size was 14 nm, 14 nm and 50 nm in x, y, and z dimensions, respectively.

Three series of computational simulations were run with MD, each for polysorbate 80 concentrations of 0.125, 0.25 and 0.5% (w/w). In the first series, simulation boxes included only tricaprylin, water, and polysorbate 80. In the second, sodium taurocholate and phosphatidylcholine were added to fully equilibrated systems from the first series. In the third series, an additional single lipase was added to the systems. Human PLRP2 was used as a model lipase (Protein Data Bank code 2OXE) to observe generic trends in the surface accessibility of the tricaprylin molecules in the presence of surfactant, bile salt and phospholipid. The trajectories were analyzed visually and with the “gmx mindist” tool of Gromacs software package. The number of contacts was counted at the standard distance of 0.6 nm between the beads, while multiple contacts with the tricaprylin group were treated as one for both water and enzyme. The data was collected over the last microsecond of the production simulation and presented as average value ± standard deviation.

## Results

### Effect of polysorbate 80 concentration on the intrinsic lipolysis rate of tricaprylin

The non-ionic surfactant polysorbate 80 was used to stabilize the acylglycerol nanoemulsions. Tricaprylin emulsions with varying polysorbate 80 concentrations (0.125–2%) were prepared and digested in vitro. The emulsion droplet size was measured by DLS after dilution of the emulsion with lipolysis medium but before initiation of lipolysis with pancreatic extract. The droplet size decreased with increasing polysorbate 80 concentration (Table 1). At 0.125% polysorbate 80, the emulsion droplets were 304 ± 10 nm, decreasing to 211 ± 4 nm at 1% polysorbate 80. Between 1 and 2% polysorbate 80, no further reduction in in emulsion droplet size was observed (*p* > 0.5).

**Fig. 1:**
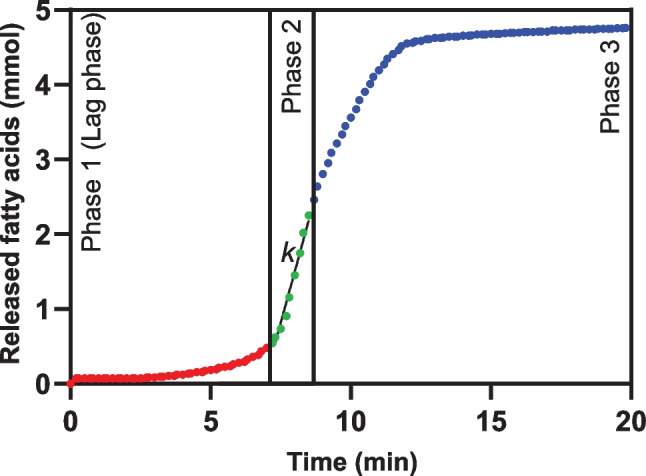
Example of a triphasic lipolysis curve. The fatty acid release during in vitro lipolysis of a tricaprylin emulsion stabilized with 1% polysorbate 80. In phase 2, *k* is the initial slope of the lipolysis curve at the point where the fatty acid release is at its highest rate, *k* is used to calculate the intrinsic lipolysis rate (ILR) according to Eq. (1)

**Fig. 2:**
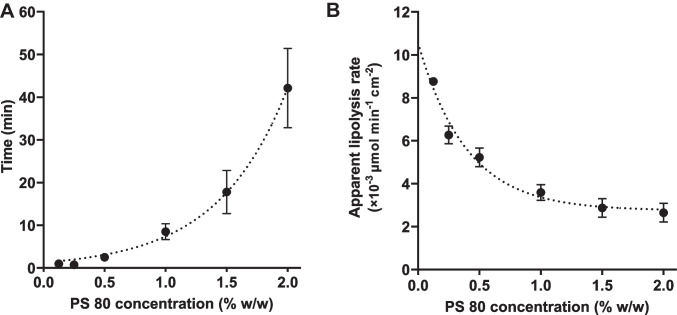
The influence of the polysorbate 80 concentration on **A** the length of the lag phase and **B** the apparent lipolysis rate of tricaprylin (*n* = 3)

Figure 1 shows the fatty acid release during the in vitro lipolysis of a tricaprylin emulsion stabilized with 1% polysorbate 80. Generally, the lipolysis of acylglycerol nanoemulsions was triphasic. In the first phase (the lag phase), fatty acid release was low and slow. In the second phase, the fatty acid release rapidly increased to its highest rate during in vitro lipolysis. The slope of the lipolysis curve in this phase (i.e., *k*) was used to calculate the apparent lipolysis rate. In the third phase, the fatty acid release decreased and then plateaued. The length of the lag phase and the slope of the lipolysis curve in the second phase, strongly depended on the polysorbate 80 concentration in the tricaprylin emulsions. The length of the lag phase increased exponentially with increasing polysorbate 80 concentration (Fig. 2a). At low polysorbate 80 (0.125–0.25%), the lag phase was short, approximately 1 min. At high polysorbate 80 (2%), the lag phase was significantly longer, exceeding 40 min. Furthermore, the apparent lipolysis rate of tricaprylin decreased with increasing polysorbate 80 concentration in the nanoemulsions (Fig. 2b). The apparent lipolysis rate was calculated following Eq. (1) for the ILR. However, the surfactant shields the lipid surface especially at high concentrations (> 0.5% polysorbate 80), thereby reducing the accessibility of the lipid surface area for digestion (Eq. 1). The apparent lipolysis rate (Fig. 2b) thus reflects the digestion of each formulation, rather than the ILR of the pure lipid.

### Effect of polysorbate 80 concentration on accessibility of tricaprylin molecules at the lipid-water interface

**Fig. 3:**
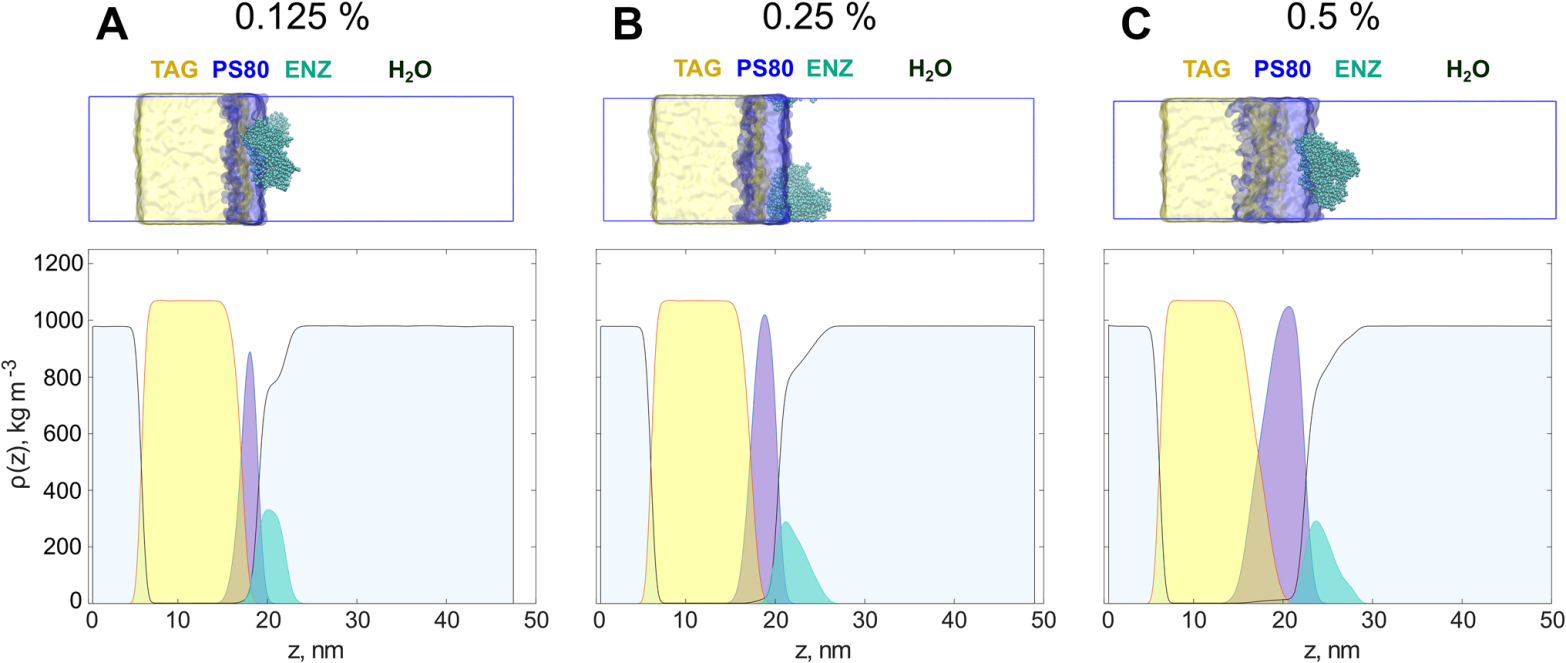
Graphical representation of the simulation boxes and corresponding density profiles of the major components. Increase of the polysorbate 80 concentration thickens the surfactant layer at the interface, resulting in an almost complete shielding of the lipids from the bulk on the right side of the box. Upper panel: graphical representation of the systems with triacylglycerol (TAG; yellow), polysorbate 80 (PS80; blue) and enzyme (ENZ; turquoise) at three surfactant concentrations: 0.125% **A**, 0.25% **B** and 0.5% **C**. Lower panel: corresponding density graphs for the components averaged over xy-planes. Colors scheme as in the upper panel

**Fig. 4:**
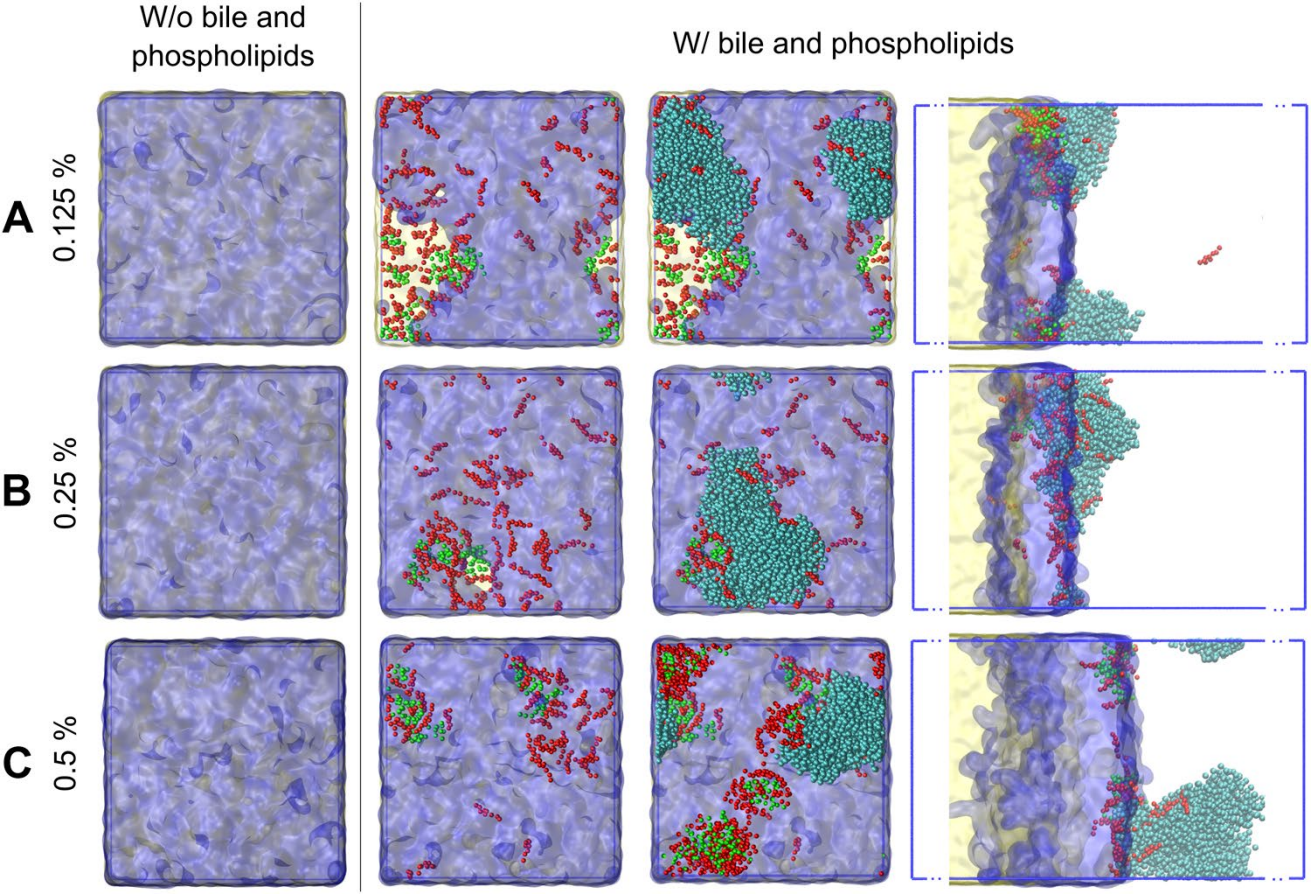
Effect of bile salts (red) and phospholipids (green) on the coverage of the tricaprylin phase (yellow) with polysorbate 80 (blue) for three polysorbate 80 concentrations: 0.125% **A**, 0.25% **B** and 0.5% **C**. The first column shows the full coverage of the right surface of the tricaprylin layer with surfactant in the absence of bile salts and phospholipids. Upon the addition of sodium taurocholate and phospholipids some polysorbate 80 molecules are expelled from the direct contact with the lipids (second column). At 0.125% polysorbate 80, a large area is occupied by sodium taurocholate and phospholipid **A**. At 0.25% polysorbate 80, this area is almost absent **B**. At 0.5% polysorbate 80, the entire surface of tricaprylin is covered with the surfactant despite significant amount of incorporated sodium taurocholate and phospholipid **C**. The enzyme (shown in the third and fourth columns in turquoise) might more easily access the surface of the lipids in the areas free from the surfactant (see the third and the fourth columns). The last column shows the simulation box from a side view, whereas first three columns are presented in top-down view

The dynamic interfacial composition of the nanoemulsions was studied by MD simulations for a mechanistic understanding of the effect of polysorbate 80 on the apparent lipolysis rate of tricaprylin (Fig. 2). All polysorbate 80 molecules formed a layer on top of the tricaprylin phase. The average thickness of this layer increased proportionally with polysorbate 80 concentration and as a result, the lipid accessibility decreased. At 0.125% polysorbate 80, both water and enzyme could reach tricaprylin through the surfactant layer (Fig. 3a). The lipid phase was nearly entirely shielded from contact with water and enzyme at 0.25% polysorbate 80 (Fig. 3b) and a complete coverage was observed at 0.5% surfactant (Fig. 3c). However, at 0.5% polysorbate 80, the layer thickness became inhomogeneous. This indicates that a partial ejection of the surfactant might take place on longer timescales (or in larger simulation boxes where boundary conditions do not enhance the flattening of the layer). Polysorbate 80 was partially expelled from the surface of tricaprylin as sodium taurocholate and phosphatidylcholine were introduced in the system (Fig. 4). As a result, surfactant-free areas formed on the lipid surface. This effect was pronounced at 0.125% (Fig. 4a), barely visible at 0.25% (Fig. 4b) and absent at 0.5% polysorbate 80 (Fig. 4c). Thus, at low surfactant concentration, sodium taurocholate and phosphatidylcholine expose tricaprylin to molecules in the continuous phase. Enzyme was most often found at the bile-rich regions of the surface (at 0.125 and 0.25% polysorbate 80) suggesting that tricaprylin would be accessible to lipase in these surfactant-free regions.

**Fig. 5:**
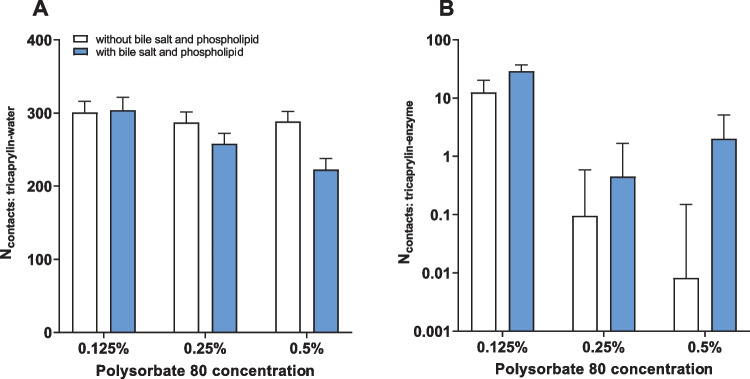
Accessibility of the tricaprylin layer covered with polysorbate 80, bile salts and phospholipids at different polysorbate 80 concentrations. **A** The average number of contacts between tricaprylin and water beads, calculated over the last microsecond of the simulation. **B** The number of contacts between tricaprylin beads and the enzyme. Only unique pairs of contacts were counted between the molecules (i.e., not for each atom of each of the molecules)

These qualitative observations were confirmed by analyzing the number of contact points between tricaprylin and water (Fig. 5a) or tricaprylin and enzyme (Fig. 5b). In general, the accessibility of tricaprylin decreased with increasing polysorbate 80 concentration. The number of contact points for both water and enzyme increased in the presence of sodium taurocholate and phosphatidylcholine at 0.125% polysorbate 80. As surfactant concentration increased, the joint layer of polysorbate 80, sodium taurocholate and phosphatidylcholine increased, shielding water molecules from tricaprylin. The number of water molecules in contact with tricaprylin was similar for 0.25 and 0.5% polysorbate 80 (in the absence of sodium taurocholate and phosphatidylcholine), confirming full coverage of the lipid surface at these surfactant concentrations. Enzyme, on the other hand, showed a higher affinity for sodium taurocholate and phosphatidylcholine compared to surfactant only. Thus, the enzyme incorporated deeper into the layer of bile salt, phospholipids and surfactant, as the concentration of the polysorbate 80 increased from 0.25 to 0.5%. Nevertheless, the overall tricaprylin accessibility was lower at these surfactant concentrations compared to 0.125% polysorbate 80 as surfactant-free regions were not formed.

### Intrinsic lipolysis rate of acylglycerols

Nanoemulsions of pure acylglycerols were all prepared at 0.25% polysorbate 80 to allow for direct comparison of their ILRs. At this surfactant concentration, the emulsion droplets achieved a hydrodynamic diameter of 200–400 nm for all lipids (Table 1). The low standard deviation and polydispersity index (< 0.3) indicated that the preparation of nanoemulsions by ultrasonication reproducibly yielded monodisperse droplets. The hydrodynamic diameter of the nanoemulsions increased with increasing acyl chain length. Thus, tricaprylin (C8:0) formed the smallest droplets and triolein (C18:1) and trilinolein (C18:2) formed the largest droplets (Table 1). The differences in size were significant in all cases except when comparing tricaprin (C10:0) and trilaurin (C12:0) (*p* = 0.07). The droplets of 1,3 dilaurin (C12:0) emulsions was in the same range as tricaprin and trilaurin (*p* > 0.5 in both cases). The droplets of trilinolein emulsions were smaller than those of the triolein emulsions (*p* = 0.015), implying that the presence of double bonds influenced the final droplet size. Emulsions of 1-monoacylglycerols and 0.5–4% polysorbate 80 phase separated or gelated (data not shown) and were therefore not included in the ILR analysis.

**Fig. 6:**
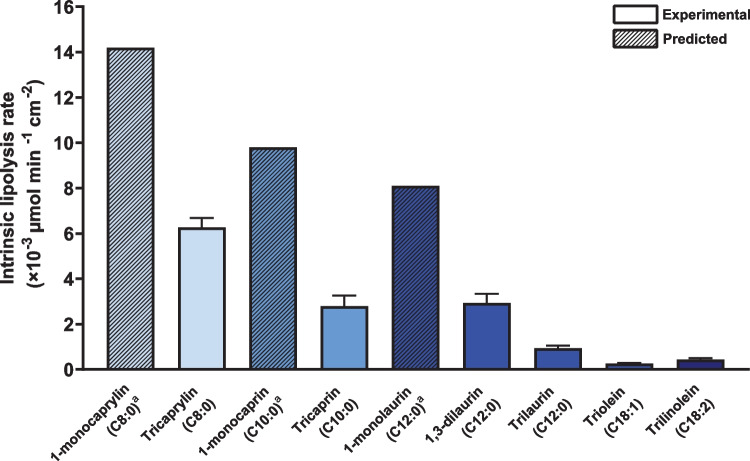
The intrinsic lipolysis rate of pure acylglycerols (purity > 95%) differing in acyl chain length, esterification, and unsaturation (*n* = 3). ^a^The ILR of 1-monoacylglycerols (i.e., 1-monocaprylin, 1-monocaprin and 1-monolaurin) is predicted from the ILR of binary acylglycerol nanoemulsions containing small amounts of 1-monoacylglycerol with trilaurin as the main component. Solid and dashed bars indicate experimental and predicted values, respectively

Lipolysis curves of all acylglycerol nanoemulsions are presented in the supplementary material (Figs. S6-S9). The ILR of pure acylglycerols differing in acyl chain length, esterification, and unsaturation are presented in Fig. 6 and Table 1. The fraction of ionized fatty acids decreased with increasing acyl chain length (see Table S6). The ILR of triacylglycerols decreased with increasing acyl chain length (*p* < 0.0013 in all cases). Thus, tricaprylin (C8:0) had the highest ILR (6.3 ± 0.3 × 10^−3^ µmol min^−1^ cm^−2^) and triolein (C18:1) had the lowest ILR (0.26 ± 0.03 × 10^−3^ µmol min^−1^ cm^−2^), i.e., 20-fold lower than tricaprylin. At the same acyl chain length, the ILR of a 1,3-diacylglycerol (1,3-dilaurin) was threefold higher than that of a triacylglycerol (trilaurin) (*p* = 0.0006). Furthermore, the ILR of a triacylglycerol with two double bonds (trilinolein) was approximately twofold higher than that of a monounsaturated triacylglycerol of the same acyl chain length (triolein) (p = 0.0033). Since 1-monoacylglycerols did not form emulsions with well-defined droplets on their own, it was not possible to determine the ILR of 1-monoacylglycerols directly. Instead, the ILR of 1-monoacylglycerols was predicted from the ILR of binary acylglycerol emulsions as presented in the next section.

### Predictions based on the intrinsic lipolysis rate

**Fig. 7:**
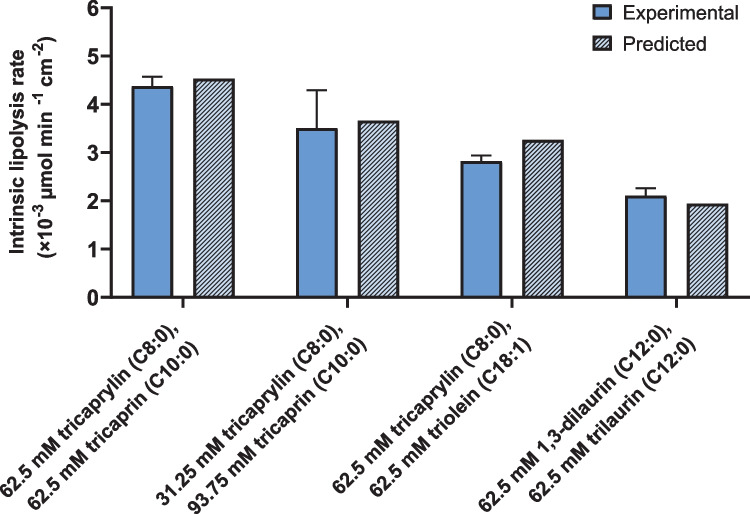
Comparison of the experimental and the predicted ILR of binary mixtures of acylglycerols. The experimental mixed ILR (open bars) is presented as the mean ± SD (*n* = 3). The predicted mixed ILR (dashed bars) is calculated based on the mean ILR of the pure components using Eq. (7)

Equation (7) suggests that the ILR of a nanoemulsion comprised of a mixture of acylglycerols can be predicted from the IRL of the respective pure components. As proof of concept, the ILRs of four binary acylglycerol nanoemulsions were determined experimentally and compared to the predicted values based on the ILRs of the individual emulsion constituents by using Eq. (7) (Fig. 7, Table 1). The experimental and predicted ILRs of the binary nanoemulsions differed by 3 (for 62.5 mM tricaprylin and 62.5 mM tricaprin) to 15% (for 62.5 mM tricaprylin and 62.5 mM triolein). On average, the experimental and predicted ILR differed by 8%, which is in the range of the expected experimental variability. The average relative standard deviation was 10%, based on all lipolysis experiments conducted with pure and binary nanoemulsions at 0.25% polysorbate 80. Overall, this suggests that formulation scientists can use the ILRs of pure components to predict the lipolysis rate of binary nanoemulsions.

Equation (7) can be further applied to predict the ILR of pure lipids for which the ILR cannot be determined experimentally. Pure lipids that do not form a stable nanoemulsion on their own, can be incorporated in an emulsion containing another lipid with a known ILR. Rearrangement of Eq. (7) allows prediction of the unknown ILR. Here, 1-monocaprylin, 1-monocaprin and 1-monolaurin did not form stable nanoemulsions on their own. However, binary emulsions of 1-monoacylglycerols and triacylglycerols (trilaurin and triolein) with a low content of 1-monoacylglycerol (i.e., molar ratio of fatty acids from triacylglycerol and monoacylglycerol 9:1 and 19:1) were stable, with droplet sizes of 200–300 nm (Table 1). Compared to the nanoemulsions containing only trilaurin or triolein, small amounts of 1-monoacylglycerol decreased the resulting emulsion droplet size. The presence of small amounts of 1-monocaprylin and 1-monocaprin significantly increased the ILR (*p* < 0.0001 and *p* = 0.03, respectively) compared to that of pure trilaurin (0.9 ± 0.09 × 10^−3^ µmol min^−1^ cm^−2^). For example, the ILR of a binary emulsion containing 25 mM 1-monocaprylin and 112.5 mM trilaurin was 2.3 ± 0.2 × 10^−3^ µmol min^−1^ cm^−2^ (Fig. 6, Table 1). In the presence of small amounts of 1-monolaurin the mixed ILR was higher than that of pure trilaurin, but the difference was not significant (*p* = 0.07). The ILRs of the binary nanoemulsions were used to predict the ILRs of 1-monoacylglycerols (Fig. 6, Table S6).

To validate the approach and confirm that the predicted 1-monoacylglycerol ILRs did not depend on the triacylglycerol component of the emulsion, an additional 1-monoacylglycerol-triacylglycerol binary emulsion was studied. This binary emulsion consisted of 12.5 mM 1-monocaprin and 118.75 mM triolein. The ILR of this mixture was 0.7 ± 0.07 × 10^−3^ µmol min^−1^ cm^−2^, which was significantly higher than that of pure triolein (*p* = 0.0009). Based on the experimental data from the trilaurin- and triolein-based binary nanoemulsions, the predicted ILR of 1-monocaprin was 9.8 × 10^−3^ µmol min^−1^ cm^−2^ and 9.3 × 10^−3^ µmol min^−1^ cm^−2^, respectively. Thus, the predicted ILRs of 1-monocaprin differed by 5%, well within the range of the expected experimental variability. Finally, comparing the predicted ILR of 1-monoacylglycerols with the experimental ILR of the 1,3-diacylglycerol and triacylglycerols of the same chain length, the 1-monoacylglycerols had the highest ILRs in all cases (Fig. 6).

## Discussion

In this study, we introduced the ILR as a simple but efficient mean to directly compare the digestibility of lipids. The surface area independent ILR was obtained from in vitro lipolysis of nanoemulsions stabilized with polysorbate 80 and was calculated by adapting the methodology developed for the suspension-based intrinsic dissolution rate [36, 37]. The IRL methodology can be combined with the previously published tool to predict the drug loading in LBFs [46]. Drug solubility and lipolysis rate can thus be determined for lipid excipients, in order to predict LBFs with best loading capacity and digestibility for a particular drug delivery system. In other words, both thermodynamics and kinetics of LBFs can be captured and give insights into formulation performance early on in the dosage form design process.

### Formulation considerations for intrinsic lipolysis rate determination

Preparation of nanoemulsions with controlled droplet sizes, and thereby a specific surface area available for digestion, was essential for determining the ILR. Polysorbate 80 was used to stabilize the nanoemulsions as this surfactant is a common pharmaceutical excipient (e.g., in Mycapssa [7]). Like other surfactants, polysorbate 80 inhibits the digestion of triacylglycerols catalyzed by pancreatic lipase, as reported for other, albeit more complex, systems [24]. This gives a good foundation for comparison between our study on a very simple system (consisting of pure tricaprylin and polysorbate 80) and previous studies on more complex systems (consisting of lipid mixtures, vegetable oil or LBF excipients, and several surfactants) [24, 25, 27]. Figure 2 shows that polysorbate 80 resulted in a decrease in lipolysis rate of tricaprylin. Specifically, increasing polysorbate 80 concentration increased the length of the lag phase exponentially and decreased the apparent lipolysis rate of tricaprylin significantly. In previous studies including medium- and long-chain triacylglycerols, polysorbate 80 inhibited lipolysis in a similar manner as observed in this study [24–27].

The inhibitory effect of polysorbate 80 on lipolysis can be explained by at least two mechanisms. Polysorbate 80 itself is a substrate for lipases because it contains esters of oleic acid, which can be cleaved by pancreatic enzymes [25, 47]. At low concentrations, polysorbate 80 inhibits lipolysis competitively. However, at higher concentrations, it inhibits lipolysis in a non-competitive manner [25]. This inhibitory mechanism can explain the retardation of the onset of lipolysis (i.e., the increase in the length of the lag phase) and can be attributed to the interfacial structure of the emulsion droplets. The interfacial structure of the lipid droplets is crucial because adsorption of the lipase to the lipid surface is a prerequisite for lipolysis. In systems containing several amphiphilic constituents (e.g., lipase, colipase, bile salts and surfactants), the adsorption of lipase to the lipid surface is a complex multi-step process. As reviewed by Golding and Wooster, bile salts and surfactants covering the lipid surface can inhibit the adsorption of lipases [48]. In contrast, the lipase–colipase complex is able to adsorb to the lipid surface even in the presence of bile salts. However, the presence of small molecule surfactants such as polysorbate 80 can restrict the adsorption of the lipase–colipase complex. In this case, the presence of bile salts, which can displace surfactants from the lipid surface, can facilitate the adsorption of the lipase–colipase complex [48]. Yao and co-workers have measured the amount of lipase adsorbed to the lipid droplet surface during in vitro lipolysis. They found that the amount of adsorbed lipase increases gradually over time until a plateau is reached, which coincides with the length of the lag phase [26].

We used MD simulations to study the interfacial structuring of a system containing one pure acylglycerol (tricaprylin), one surfactant (polysorbate 80) and one bile salt (sodium taurocholate). In agreement with the experimental data (see discussion above), the simulations showed that increasing polysorbate 80 concentration inhibited lipolysis. At low surfactant concentration (0.125% w/w), the enzyme can reach the tricaprylin surface relatively easily (Fig. 3a). Already 0.25% polysorbate 80 is sufficient to cover the acylglycerol surface, as the number of contacts between water and acylglycerol remained unaffected when increasing the surfactant concentration to 0.5% (Fig. 5b). However, in the presence of bile salt and phospholipid, a surfactant-free region was observed at 0.125% polysorbate 80, causing increased accessibility of tricaprylin for the lipase (Figs. 4, 5b). As the thickness of the polysorbate 80 layer increased with higher concentrations of it, the surfactant displacement was either small or absent. This observation is in a good agreement with the experiments (Fig. 2), in which 0.125% and 0.25% polysorbate 80 had the strongest effect on lag time and apparent lipolysis rate.

To ensure comparability and limit inhibition of lipolysis, we determined the ILR of the pure acylglycerols formulations at the same, low polysorbate 80 concentration. The polysorbate 80 concentration during lipolysis was 0.25% for all nanoemulsions, which was the lowest concentration at which all acylglycerols formed nanoemulsions via ultrasonication (i.e., a 0.5% polysorbate 80 solution was used for preparation). The MD simulations indicated almost full coverage of the acylglycerol layer with polysorbate 80 at 0.25% although a thinner layer was formed as compared to 0.5%. Still, it should be noted that due to computational constraints, MD simulations can only simulate the interfacial structure on a very short time scale (3 microseconds). Replacement of polysorbate 80 molecules by bile salt and phospholipids, which would make the acylglycerol accessible for the enzyme, likely happens at longer time scales.

The physical state of the lipid (liquid *vs.* solid) also influences lipolysis. Liquid lipids are digested more readily than solid lipids [49]. To prepare the nanoemulsions of solid acylglycerols, the acylglycerols were melted and immediately ultrasonicated. With this process, we assume that we obtained emulsions of supercooled melts and thus that all acylglycerols were in a liquid state in the study. Bunjes and co-workers report that emulsions of supercooled melted trilaurin do not crystallize within 5 months of storage even below room temperature [50].

### Intrinsic lipolysis rate of pure lipid excipients for early dosage form design

The ILR approach is analytically and mathematically simple facilitating its facile use during drug development. The ILR method makes no assumptions, mathematically, about the mechanism of action for the mass transport process. A linear model is fit to the fatty acid concentration versus time data of the initial release phase. As long as a linear model is found for a set of times after the lag phase, this is accepted as the “intrinsic” range for the calculation. We also compared lipolysis rates determined using the first-order mechanistic model described by Li and McClements and Gaucel et al. to justify the correctness of the assumptions made to calculate the ILR [31, 32]. The mechanistic model estimates the rate constant assuming a monodisperse population of spherical oil droplets with full access to the digestion medium. Additionally, the model assumes that this population of identically shaped spheres changes size uniformly with time as lipids are digested, resulting in the increase in the fraction digested. Thus, the monodisperse diameter of the lipid droplets decreases with time. The rate constant is determined by a least squares regression estimation, by iteratively guessing the rate constant and extent of digestion until the root mean square error is maximally minimized. However, other factors that influence lipid droplet size evolution are not accounted for by the mechanistic model, such as droplet polydispersity, aggregation, phase separation and interfacial structuring of components (as demonstrated here by the MD simulations). It may be that these factors account for the deviation in the mechanistic model for the predicted fraction versus in vitro lipolysis data of the pure acylglycerols (Figs. S1-S3). The IRL and mechanistic model predicted similar digestion rate constants (Table 1). This supports the validity of the IRL methodology and demonstrates that it is a simple and predictive tool for designing the digestion of LBFs.

We here compared the digestibility of nine pure acylglycerols differing in acyl chain length, esterification, and unsaturation. For this, equimolar amounts of acylglycerols were digested (rather than the same mass or volume) as this provides a more mechanistically informative assessment of the activity of pancreatic lipase on different substrates [51]. As expected, the ILR of triacylglycerols decreased with increasing acyl chain length. It is well described that medium-chain lipids are digested more readily than long-chain lipids [51]. According to Benito-Gallo and co-workers, the decrease in lipolysis with increasing acyl chain length can be explained by: the droplet size of the triacylglycerol emulsions, the solubility of the 2-monoacylglycerols within mixed micelles and/or the relative stability of the fatty acids as leaving groups in the hydrolysis reaction [51]. By design, the ILR facilitates the comparison of lipids independent of droplet size. Thus, the solubility of the 2-monoacylglycerols and/or the relative stability of the fatty acids as leaving group explain the chain length effect rather than the emulsion droplet size. During lipolysis, 2-monoacylglycerols accumulate at the lipid-water interface, which can inhibit lipase adsorption. Solubilization in mixed micelles can remove 2-monoacylglycerols from the interface and counteract their inhibitory effect. The ILR of trilaurin was threefold lower than that of 1,3-dilaurin. Only during the lipolysis of trilaurin, 2-monolaurin is formed. In contrast, lipolysis of 1,3-dilaurin yields two fatty acids and free glycerol. The absence of inhibitory 2-monolaurin can explain why the ILR of 1,3-dilaurin was higher than that of trilaurin, which is consistent with Benito-Gallo and co-workers’ hypothesis.

The influence of fatty acid unsaturation on lipid digestion has previously not been studied to the same extent as the fatty acid chain length. Triolein and trilinolein have the same acyl chain length and esterification, but differ in degree of unsaturation (i.e., one *vs.* two double bonds). The ILR of triolein (C18:1) was approximately half of that of trilinolein (C18:2). Pascoviche and co-workers report that olive oil (rich in oleic acid) is more susceptible to hydrolysis than hempseed oil (rich in linoleic acid) and pomegranate seed oil (rich in linolenic acid) [52]. In contrast to the current study on pure lipids, the digestibility of vegetable oils thus decreased with increasing unsaturation. The composition of the vegetable oils, which are mixtures of different acylglycerols, can explain the inconsistency between our and their study. Analysis of the fatty acid composition showed that olive oil contains significantly more palmitic acid (C16:0; ~ 10%) than hempseed oil (~ 5%) and pomegranate oil (~ 2%) [52]. Here, we clearly showed that lipolysis increased with decreasing fatty acid acyl chain length, and we showed that the lipolysis of lipid mixtures depended on the composition of the mixture (discussed below). The difference in palmitic acid content of the vegetable oils may therefore have had a significant impact on the lipolysis. The effect of unsaturation on lipid digestion deserves further studies, preferably on pure lipids.

### Intrinsic lipolysis rate as a predictive formulation tool

Nanoemulsions of acylglycerols mixtures were digested in vitro and an ILR calculated for the binary formulations. Clearly, the mixed ILR depended on the composition of the mixture. We showed that the ILR of binary nanomemulsions could be predicted from the ILR of the pure components using Eq. (7) (Fig. 7). Even though previous studies have determined digestion rate constants of oils and triacylglycerols by applying a first-order reaction model to pH–stat lipolysis data [31, 33, 35], these constants have not previously been used to predict the digestion rate constant of lipid mixtures. Giang and co-workers determined the individual lipolysis rates of fatty acids with various chain lengths from a lipid mixture that was digested in vitro. As a prerequisite for this approach, the decrease of medium-chain and long-chain triacylglycerols during digestion is monitored via HPLC connected to an evaporative light scattering detector and the formation of fatty acids monitored via gas chromatography [53]. Since it is not possible to distinguish between different fatty acids released during lipolysis via titration, these analytical methods are required to obtain the individual lipolysis rates. In reverse, here we determined the ILR of pure acylglycerols via simple titration (i.e., pH–stat lipolysis) and used the individual lipolysis rates to predict the rates of their mixtures.

A prerequisite for determining the ILR is emulsification of the lipid to obtain a controlled surface area available for digestion. Not all lipid excipients can form emulsions. Because of their amphiphilic nature, it was not possible to prepare emulsions of 1-monoacylglycerols; these exhibit a complex phase behavior in water [54]. Hence, an ILR of 1-monoacylglycerols could not be determined directly. Since the mixed ILR is an expression of the ILR of the single components, we predicted the ILR of 1-monoacylglycerols from the ILRs of binary nanoemulsions by rearranging Eq. (7). For this purpose, nanoemulsions were prepared based on trilaurin and triolein and that contained small amounts of 1-monoacylglycerol. The predicted ILR suggested that 1-monoacylglycerols are digested faster than corresponding triacylglycerols and 1,3-diacylglycerols.

It should be noted that the literature does not always specify which monoacylglycerol isomer (sn-1(3) *vs*. sn-2) is present and the isomer type affects digestibility. The 2-monoacylglycerols, which are formed during digestion of triacylglycerols, are digested much slower than triacylglycerols [55]. The digestion of 2-monoacylglycerols depends on the isomerization to the sn-1(3) isomer for digestion by the sn-1(3) specific pancreatic lipase or the presence of other lipases that can cleave ester bonds at the sn-2 position (e.g., PLRP2). In contrast, 1(3)-monoacylglycerols with a sn-1(3) ester bond are accessible for pancreatic lipase. High digestion rates for partial acylglycerols have been reported [56–58]. For example, Martin and co-workers report that 1(3)-monoolein digests faster than diolein and triolein, consistent with the ILRs reported here. 1-monoacylglycerols are thermodynamically more stable than 2-monoacylglycerols [59] and therefore the sn-1(3) isomer should be the dominating species in lipid excipients containing monoacylglycerols (e.g., Capmul MCM contains medium-chain monoacylglycerols and Maisine CC contains long-chain unsaturated mono- and diacylglycerols). Unfortunately, the isomeric composition of lipid excipients is not readily available.

We here demonstrated the potential of the ILR approach to predict the in vitro digestibility of nanoemulsions. The experimental conditions of the LCFS in vitro lipolysis method, which was used to experimentally determine the ILR, employs experimental conditions resembling in vivo conditions (e.g., in vivo relevant bile salt and enzyme concentrations) [19]. However, the gastrointestinal environment in vivo is clearly more complex and dynamic. The composition of human gastrointestinal fluids varies along the gastrointestinal tract, with disease state and between individuals [60]. This can influence the size and shape of colloidal assemblies present in vivo, which in turn influences the digestion rate. Information on the composition and ultrastructure of gastrointestinal fluids can be obtained from aspirated gastrointestinal fluids [61]. The ultrastructure can be analyzed using imaging techniques (e.g., cryo-TEM) [62] or scattering techniques (e.g., AF4-MALLS) [63]. Those techniques could help clarify the in vitro in vivo correlation of the ILR approach. Nevertheless, as the ILR is normalized by surface area, it offers formulation scientist a mechanistic understanding of the digestibility of lipid excipients. Currently, it is primarily intended as a tool during LBF development to early on account for the influence of digestion kinetics on the performance of enabling drug delivery systems.

## Conclusions

This study introduces the ILR as a straightforward approach to compare the digestibility of lipid excipients commonly used in LBFs. Nanoemulsions of nine pure acylglycerols were formed with polysorbate 80 as emulsifier. Surfactant concentration in the formulations was optimized by experiments and MD simulations, to balance emulsion stability versus the inhibitory effect of polysorbate 80 on digestion at the lipid interface. Lipolysis data were normalized by the droplet surface area to calculate the ILR. This enabled the direct comparison of lipid digestibility of acylglycerols with varying acyl chain length, degree of esterification, and unsaturation. Furthermore, the ILR of binary acylglycerol nanoemulsions were successfully predicted using the ILR of the pure acylglycerols. Thus, the ILR approach can facilitate the systematic design of LBFs with known digestion kinetics early on during formulation development of oral dosage forms. Furthermore, it can serve as a tool in quality assurance to quantify and predict the influence of excipient batch-to-batch variability on formulation digestion and performance. Finally, insights from MD simulations on the dynamic interplay of components at the lipid interface could further support the development of more complex mathematical models for digestion of pharmaceutical LBFs.

## Supplementary Information

Below is the link to the electronic supplementary material.

Supplementary file1 (DOCX 2672 KB)

## Data Availability

The datasets generated during the current study are available in the SciLifeLab Data repository, https://doi.org/10.17044/scilifelab.20310249.
