# Supplementary material for: Intrinsic lipolysis rate for systematic design of lipid-based formulations
Source: Drug Deliv Transl Res. 2022 Oct 8;13(5):1288–304. doi: 10.1007/s13346-022-01246-y (PMC10102029; doi:10.1007/s13346-022-01246-y)
Supplement: Supplementary file 1 — Supplementary file1 (DOCX 2672 KB) [file 13346_2022_1246_MOESM1_ESM.docx]

Supplementary material:

Intrinsic lipolysis rate for systematic design of lipid-based formulations

Ann-Christin Jacobsen^1^, Aleksei Kabedev^2^, Patrick D. Sinko^2^, Johan E. Palm^3^, Christel A.S. Bergström^1^ and Alexandra Teleki^1,4*^

^1^The Swedish Drug Delivery Center, Department of Pharmacy, Uppsala University, Uppsala, Sweden

^2^Department of Pharmacy, Uppsala University, Uppsala, Sweden

^3^Oral Product Development, Pharmaceutical Technology & Development, Operations, AstraZeneca, Gothenburg, Sweden

^4^Department of Pharmacy, Science for Life Laboratory, Uppsala University, Uppsala, Sweden

***Corresponding author:**

Alexandra Teleki, PhD

Associate Professor

Science for Life Laboratory
Department of Pharmacy

Uppsala University

Box 580, SE-75123 Uppsala, Sweden

**Email:** [alexandra.teleki@scilifelab.uu.se](mailto:alexandra.teleki@scilifelab.uu.se)

**Phone:** +46 – 18 471 47 45

**Submitted to:** *Drug Delivery and Translational Research, CRS Local Chapter Special Issue*

**Date:** July 2022

Keywords: Lipid digestion, nanoemulsion, lipid-based formulations, molecular dynamics simulations, lipolysis, drug development.

**Table S1** Structure and properties of acylglycerols

| **Acylglycerol** | **Acyl chain length** | **Number of ester bonds** | **Number of double bonds** | **Molecular weight (g/mol)** | **Melting point (°C)** | **Purity (%)** |
| --- | --- | --- | --- | --- | --- | --- |
| 1-monocaprylin | 8 | 1 | 0 | 218.3 | 39-43 | 98 |
| Tricaprylin | 8 | 3 | 0 | 470.7 | 9 | 99 |
| 1-monocaprin | 10 | 1 | 0 | 246.3 | 51-55 | 98 |
| Tricaprin | 10 | 3 | 0 | 554.8 | 31-35 | 98 |
| 1-monolaurin | 12 | 1 | 0 | 274.4 | 60-66 | 98 |
| 1,3-dilaurin | 12 | 2 | 0 | 456.7 | 56-61 | 96 |
| Trilaurin | 12 | 3 | 0 | 639.0 | 44-49 | 98 |
| Triolein | 18 | 3 | 1 | 885.5 | 5 | 95 |
| Trilinolein | 18 | 3 | 2 | 879.4 | -5 - -4 | 95 |

**Table S2** Composition of nanoemulsions, the density used to calculate the total surface area available for digestion and total ultrasonication time used for nanoemulsion preparation.

| **Acylglycerol(s) in nanoemulsion** | **Density (g/cm^3^)** | **Polysorbate 80 concentration (%; w/w)** | **Ultrasonication time (min)** |
| --- | --- | --- | --- |
| ***Nanoemulsions with constant tricaprylin content but varying Polysorbate 80 content*** | | | |
| 125 mM tricaprylin (C8:0) | 0.938^a^ | 0.125 | 0.5 |
|  |  | 0.25 |  |
|  |  | 0.5 |  |
|  |  | 1 |  |
|  |  | 1.5 |  |
|  |  | 2 |  |
| ***Nanoemulsions of pure acylglycerols with constant Polysorbate 80 content*** | | | |
| 125 mM tricaprin (C10:0) | 0.922^a^ | 0.25 | 1 |
| 125 mM 1,3 dilaurin (C12:0) | 0.953^b^ |  | 1 |
| 125 mM trilaurin (C12:0) | 0.9085^c^ |  | 1 |
| 125 mM triolein (C18:1) | 0.8991^c^ |  | 5 |
| 125 mM trilinolein (C18:2) | 0.913^c^ |  | 5 |
| ***Binary nanoemulsions of pure acylglycerols with constant Polysorbate 80 content*** | | | |
| 62.5 mM tricaprylin (C8:0), 62.5 mM tricaprin (C10:0) | 0.930^d^ | 0.25 | 0.5 |
| 31.25 mM tricaprylin (C8:0), 93.75 mM tricaprin (C10:0) | 0.926^d^ |  | 0.5 |
| 62.5 mM tricaprylin (C8:0), 62.5 mM triolein (C18:1) | 0.919^d^ |  | 5 |
| 62.5 mM 1,3 dilaurin (C12:0), 62.5 mM trilaurin (C12:0) | 0.931^d^ |  | 1 |
| 112.5 mM trilaurin (C12:0), 25 mM 1-monocaprylin (C8:0) | 0.9085^e^ |  | 1 |
| 118.75 mM trilaurin (C12:0), 12.5 mM 1-monocaprin (C10:0) | 0.9085^e^ |  | 1 |
| 118.75 mM trilaurin (C12:0),12.5 mM 1-monolaurin (C12:0) | 0.9085^e^ |  | 1 |
| 118.5 mM triolein (C18:1), 12.5 mM 1-monocaprin (C10:0) | 0.8991^e^ |  | 5 |

^a^ Density at 37 °C taken from Goodrum and Eiteman 1996 *Bioresource Technology* 56, [DOI: 10.1016/0960-8524(95)00167-0](https://doi.org/10.1016/0960-8524(95)00167-0).
^b^ Predicted value calculated using Advanced Chemistry Development (ACD/Labs) Software V11.02 (© 1994-2022 ACD/Labs).
^c^ Density at 40 °C taken from Carter Lichtfeld, Analysis of triglycerides, 1972, Chapter 11 ”Physical properties”, ISBN: 9780323153522.
^d^ Calculated from the density of the single components taking their molar ratio into account and assuming ideal mixing.
^e^ Density of the triacylglycerol (we assume that those small amounts of monoacylglycerol do not cause a significant change in density).

**Appendix S1** Executable MATLAB script for fitting multiple sets of digestion data.

clear all

close all

clc

%% Fraction of FFA release model Li and McClements, J. Agric. Food Chem., Vol. 58, No. 13, 2010

%phi(i) = phi_max.*(1-(1+((3.*k.*M.*t)./(2.*d0.*p))).^-2);

% Corrected model J. Agric.Food Chem., 2015, 63, 10352-10353

%phi(i) = phi_max.*(1-(1-((k.*M.*t)./(d0.*p))).^3);

%% Import

fprintf('Select Constants data file \n')

[name, path] = uigetfile('*.xlsx','Select file to open');

%file: full path of the text file

file = [path, name];

[num,txt,raw] = xlsread(file);

data_constant = num; % numerical data from excel file

labels_constants = txt; % headers from excel file

M = data_constant(1,:); %assigning molecular weight to variable

d0 = data_constant(2,:); %assigning initial droplet size to variable

p = data_constant(3,:); %assigning oil density to initial variable

fprintf('Select Time Course data file \n')

[name, path] = uigetfile('*.xlsx','Select file to open');

%file: full path of the text file

file = [path, name];

[num_exp,txt_exp,raw_exp] = xlsread(file);

data_exp = num_exp; % numerical data from excel file

labels_constants = txt_exp; % headers from excel file

t = data_exp(:,1);

%extracting the time series response data from data set

%(all but left most colum which is time data)

f = zeros(length(data_exp),min(size(data_exp))-1);

for i=2:1:min(size(data_exp))

f(:,i-1) = data_exp(:,i);

end

%% Input Constants (Change for each lipid)

for i=1:length(M)

M(i) = M(i)./1000; % molecular weight (kg/mol)

d0(i) = d0(i).*10.^-9; %initial droplet size (m)

p(i) = p(i).*(1*10.^3); % density of oil (kg/m3)

end

%% Non-linear Fitting

% b(1) = phi_max

% b(2) = k

modelfun = @(b,x)b(1).*(1-max(0,(1-((b(2).*M(i).*x)./(d0(i).*p(i)))).^3));

beta0 = [1 1*10^-6];

opts = statset('nlinfit');

opts.RobustWgtFun = 'bisquare';

for i=1:length(M)

mdl = fitnlm(t,f(:,i),modelfun,beta0,'Options',opts); % non-linear fitting

%fn(time import, fraction import, model function to be fit, initial

%values)

phi_max_fit(i) = mdl.Coefficients{1,1}; %collecting fit phi max values

k_fit(i) = mdl.Coefficients{2,1}; %collecting fit k values

r2(i) = mdl.Rsquared.Ordinary; %collecting r^2 values

rmse(i) = mdl.RMSE;%collecting root mean square error values

end

%% Model Calculations using fit parameters

% phi_max_fit = mdl.Coefficients{1,1};

% k_fit = mdl.Coefficients{2,1};

time =(0:0.1:90);

for i=1:length(M)

for j=1:1:length(time)

model_phi(i,j) = phi_max_fit(i).*(1-max(0,(1-((k_fit(i).*M(i).*(time(j)))./(d0(i).*p(i)))).^3));

end

end

% model_phi =model_phi.'; %tranposing for consistency with imported data

% time =time.';%tranposing for consistency with imported data

%% Visulaization Check

for i=1:length(M)

figure

plot(time(:),model_phi(i,:))

hold on

plot(t(:),f(:,i),'o')

hold off

xlabel('Time')

ylabel('\Phi')

end

%% Output for post processing

%Table summary for fit absorption rate and maximum release

for i=2:1:min(size(data_exp))

labels(i-1)=labels_constants(i);

end

VarNames = {'Compound';'K_abs'; 'Max_frac_released'; 'R2'; 'RMSE'};

T1 =table(labels(:),k_fit(:),phi_max_fit(:),r2(:),rmse(:), 'VariableNames',VarNames)

**Table S3** Input Constants to Regression Model

| **Constants** | **M (g/mol)** | **d_0_ (nm)** | ***ρ_0_(g/cm^3^)*** |
| --- | --- | --- | --- |
| Tricaprylin 0.5% PS80-1 | 470.70 | 255.7 | 0.938 |
| Tricaprin 0.5% PS80-1 | 554.80 | 278.4 | 0.922 |
| Trilaurin 0.5% PS80-1 | 639.00 | 281.5 | 0.909 |
| Tricaprylin 3% PS80-1 | 470.70 | 254.0 | 0.953 |
| Triolein 0.5% PS80-1 | 885.40 | 396.3 | 0.899 |
| TriC8 TriC10 0.5% PS80-1 | 512.75 | 255.1 | 0.930 |
| TriC8 TriC10 0.5% PS80-1 | 512.75 | 307.0 | 0.919 |
| TriC8 TriC10 1to3 0.5% PS80-1 | 536.26 | 258.2 | 0.926 |
| Trilinolein 0.5% PS80-1 | 879.40 | 371.0 | 0.913 |
| trilaurin dilaurin 1to1-1 | 547.85 | 281.4 | 0.931 |


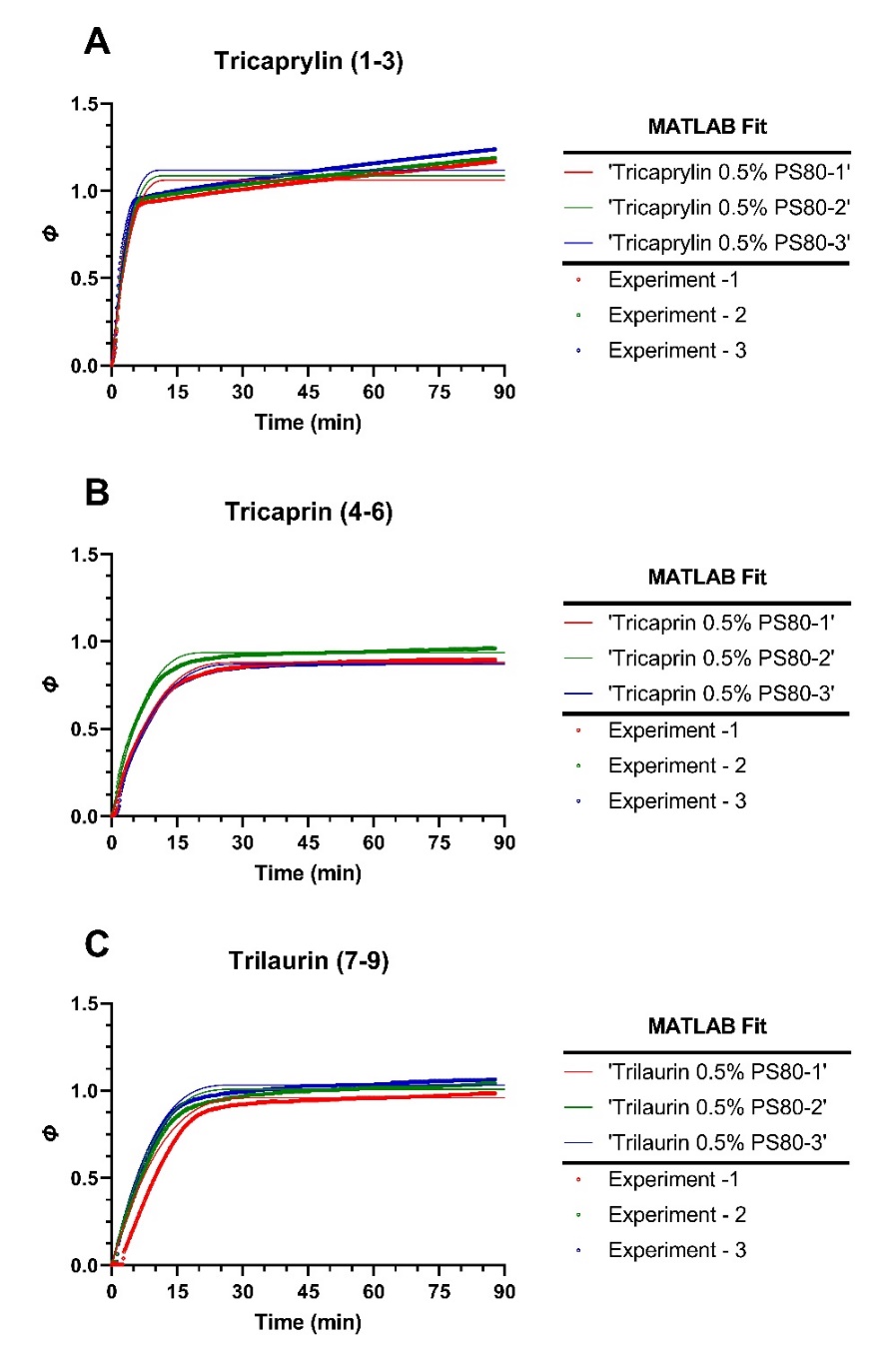


**Fig. S1** Visualizations of Non-linear Model Fitting for Lipolysis data. Lipolysis curves from the in vitro digestion of nanoemulsions containing one pure acylglycerol at a constant Polysorbate 80 content (0.25%). A) tricaprylin (C8), B) tricaprin (C10), C) trilaurin (C12). The green, red, and blue circles represent replicate 1, 2 and 3, respectively. The solid line represents the predicted digestion profile according to equation 9.


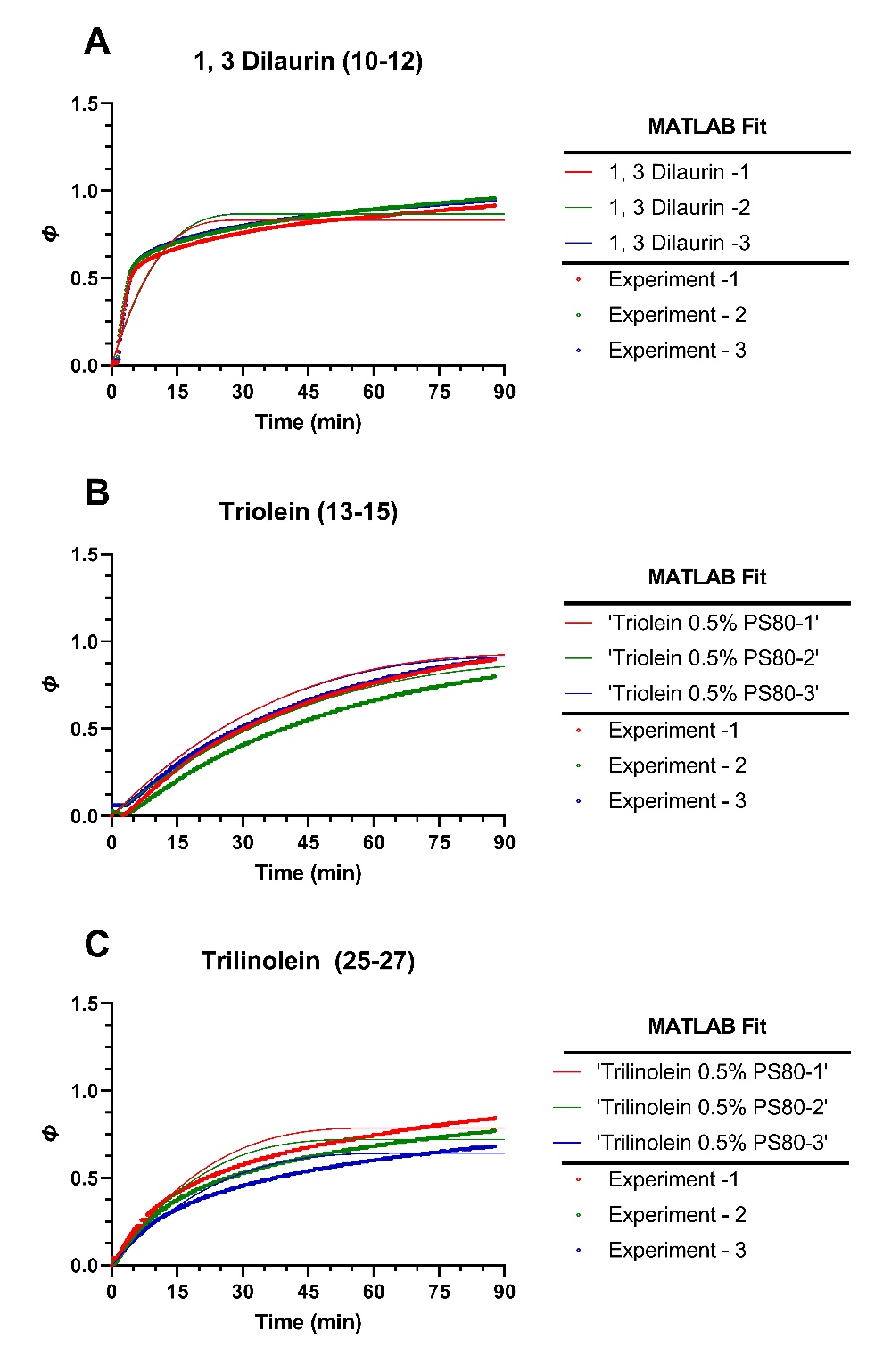


**Fig. S2** Visualizations of Non-linear Model Fitting for Lipolysis data. Lipolysis curves from the in vitro digestion of nanoemulsions containing one pure acylglycerol at a constant Polysorbate 80 content (0.25%). %). A) 1,3‑dilaurin (C12), B) triolein (C18:1) and C) trilinolein (C18:2). The green, red, and blue circles represent replicate 1, 2 and 3, respectively. The solid line represents the predicted digestion profile according to equation 9.


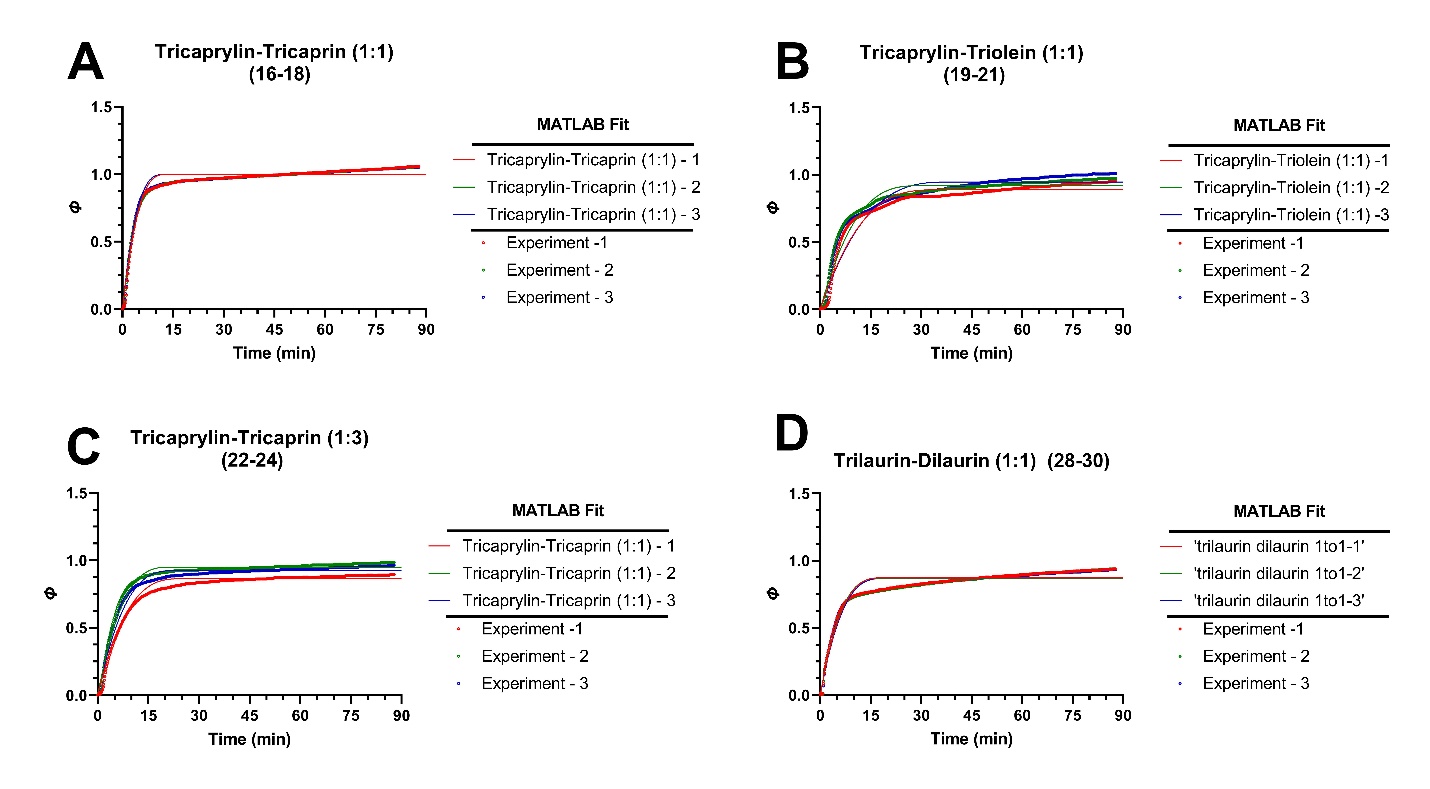


**Fig. S3** Visualizations of Non-linear Model Fitting for Lipolysis data. Lipolysis curves from the in vitro digestion of binary acylglycerol nanoemulsions at a constant Polysorbate 80 content (0.25%). A) 62.5 mM tricaprylin (C8) + 62.5 mM tricaprin (C10), B) 62.5 mM tricaprylin (C8) + 62.5 mM triolein (C18:1), C) 31.25 mM tricaprylin (C8 + 93.75 mM tricaprin (C10), and D) 62.5 mM 1,3‑dilaurin (C12) + 62.5 mM trilaurin (C12).The green, red, and blue circles represent replicate 1, 2 and 3, respectively. The solid line represents the predicted digestion profile according to equation 9.

**Fig. S4** Martini force field topologies for the polysorbate 80 (A) and tricaprylin (B) molecules. ID-numbers are specified in black colour and match those in the topology tables S5 and S6.

**Table S4** Topology parameters for tricaprylin.

[ atoms ]

**;id type resnr residu atom cgnr charge**

1 C1 1 TCAPR GLY 1 0

2 Na 1 TCAPR ES1 2 0

3 Na 1 TCAPR ES2 3 0

4 Na 1 TCAPR ES3 4 0

5 C2 1 TCAPR C1A 5 0

6 C2 1 TCAPR C2A 6 0

7 C2 1 TCAPR C1B 7 0

8 C2 1 TCAPR C2B 8 0

9 C2 1 TCAPR C1C 9 0

10 C2 1 TCAPR C2C 10 0

**Table S4** continued

[ bonds ]

**; i j funct length force.c.**

1 2 1 0.47 1250

1 3 1 0.47 1250

1 4 1 0.47 1250

2 5 1 0.47 1250

5 6 1 0.47 1250

3 7 1 0.47 1250

7 8 1 0.47 1250

4 9 1 0.47 1250

9 10 1 0.47 1250

[ angles ]

**; i j k funct angle force.c.**

2 1 4 2 60.000 25.0

2 1 3 2 130.000 25.0

3 1 4 2 130.000 25.0

1 2 5 2 180.000 25.0

2 5 6 2 180.000 25.0

1 3 7 2 180.000 25.0

3 7 8 2 180.000 25.0

1 4 9 2 180.000 25.0

4 9 10 2 180.000 25.0

**Table S5** Topology parameters for polysorbate 80.

[ atoms ]

1 C1 1 PS8 C1A 1 0

2 C2 1 PS8 C2A 2 0

3 C3 1 PS8 C3A 3 0

4 C2 1 PS8 C4A 4 0

5 C2 1 PS8 C5A 5 0

6 Na 1 PS8 ES1 6 0

7 N0 1 PS8 N7A 7 0

8 N0 1 PS8 N8A 8 0

9 N0 1 PS8 N9A 9 0

10 N0 1 PS8 N10A 10 0

11 N0 1 PS8 N11A 11 0

12 N0 1 PS8 N12A 12 0

13 N0 1 PS8 N13A 13 0

14 N0 1 PS8 N14A 14 0

15 N0 1 PS8 N15A 15 0

16 N0 1 PS8 N16A 16 0

17 P2 1 PS8 S17A 17 0

18 SN0 1 PS8 S18A 18 0

19 SN0 1 PS8 S19A 19 0

20 SN0 1 PS8 S20A 20 0

21 N0 1 PS8 N21A 21 0

22 N0 1 PS8 N22A 22 0

23 N0 1 PS8 N23A 23 0

24 N0 1 PS8 N24A 24 0

25 P2 1 PS8 S25A 25 0

26 N0 1 PS8 N26A 26 0

27 N0 1 PS8 N27A 27 0

28 N0 1 PS8 N28A 28 0

29 N0 1 PS8 N29A 29 0

30 P2 1 PS8 S30A 30 0

[bonds]

**; i j funct length force.c.**

1 2 1 0.47 1250

2 3 1 0.47 1250

3 4 1 0.47 1250

4 5 1 0.47 1250

5 6 1 0.47 1250

6 7 1 0.47 1250

7 8 1 0.47 1250

8 9 1 0.47 1250

9 10 1 0.47 1250

10 11 1 0.47 1250

12 13 1 0.47 1250

13 14 1 0.47 1250

14 15 1 0.47 1250

15 16 1 0.47 1250

16 17 1 0.47 1250

21 22 1 0.47 1250

22 23 1 0.47 1250

23 24 1 0.47 1250

24 25 1 0.47 1250

18 21 1 0.47 1250

18 11 1 0.47 1250

18 12 1 0.47 1250

18 19 1 0.47 1250

18 20 1 0.47 1250

19 20 1 0.47 1250

20 26 1 0.47 1250

26 27 1 0.47 1250

27 28 1 0.47 1250

28 29 1 0.47 1250

29 30 1 0.47 1250

[angles]

**; i j k funct angle force.c**

1 2 3 2 180.0 25.0

2 3 4 2 120.0 45.0

3 4 5 2 180.0 25.0

4 5 6 2 180.0 25.0

5 6 7 2 180.0 25.0

6 7 8 2 180.0 25.0

7 8 9 2 180.0 25.0

8 9 10 2 180.0 25.0

9 10 11 2 180.0 25.0

12 13 14 2 180.0 25.0

13 14 15 2 180.0 25.0

14 15 16 2 180.0 25.0

15 16 17 2 180.0 25.0

18 12 13 2 180.0 25.0

18 21 22 2 180.0 25.0

21 22 23 2 180.0 25.0

22 23 24 2 180.0 25.0

23 24 25 2 180.0 25.0

18 20 26 2 180.0 25.0

20 26 27 2 180.0 25.0

26 27 28 2 180.0 25.0

27 28 29 2 180.0 25.0

28 29 30 2 180.0 25.0

10 11 18 2 120.0 25.0

11 18 19 2 120.0 25.0

12 18 20 2 120.0 25.0

21 18 19 2 120.0 25.0

18 19 20 2 60.0 25.0

19 20 18 2 60.0 25.0

20 18 19 2 60.0 25.0


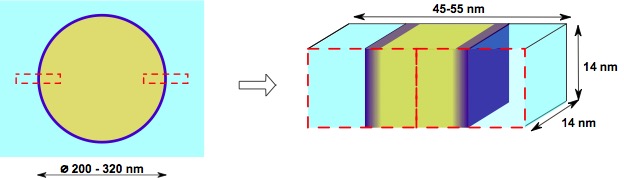


**Fig. S5** Schematic representation of the lipid droplets observed in the experiment (left part) and a corresponding representation in computer simulations (right part). We assume that the triacylglycerol phase (yellow) is homogeneous, thus there is no need to introduce the entire width of the droplet to investigate the surface structure. Surfactant molecules (violet) only stay at the surface of the lipids and their concentration in the layer of triglycerides does not depend on the width of the lipid layer. The numbers of polysorbate 80 molecules in the simulations were chosen to correspond the numbers of polysorbate 80 molecules on the surface of the actual lipid droplets, given the assumption that entire surfactant mass would be covering the surface of the lipids. That is one of the two extremums framing the real portion of the polysorbate placed at the surface of the lipid droplets (another being no surfactant molecules are covering the lipid droplets). As we are only using the area with the side length of 14 nm, curvature of the actual colloid is negligible. Bile salts and phospholipids were added at concentrations of 3 mM and 0.75 mM, respectively.


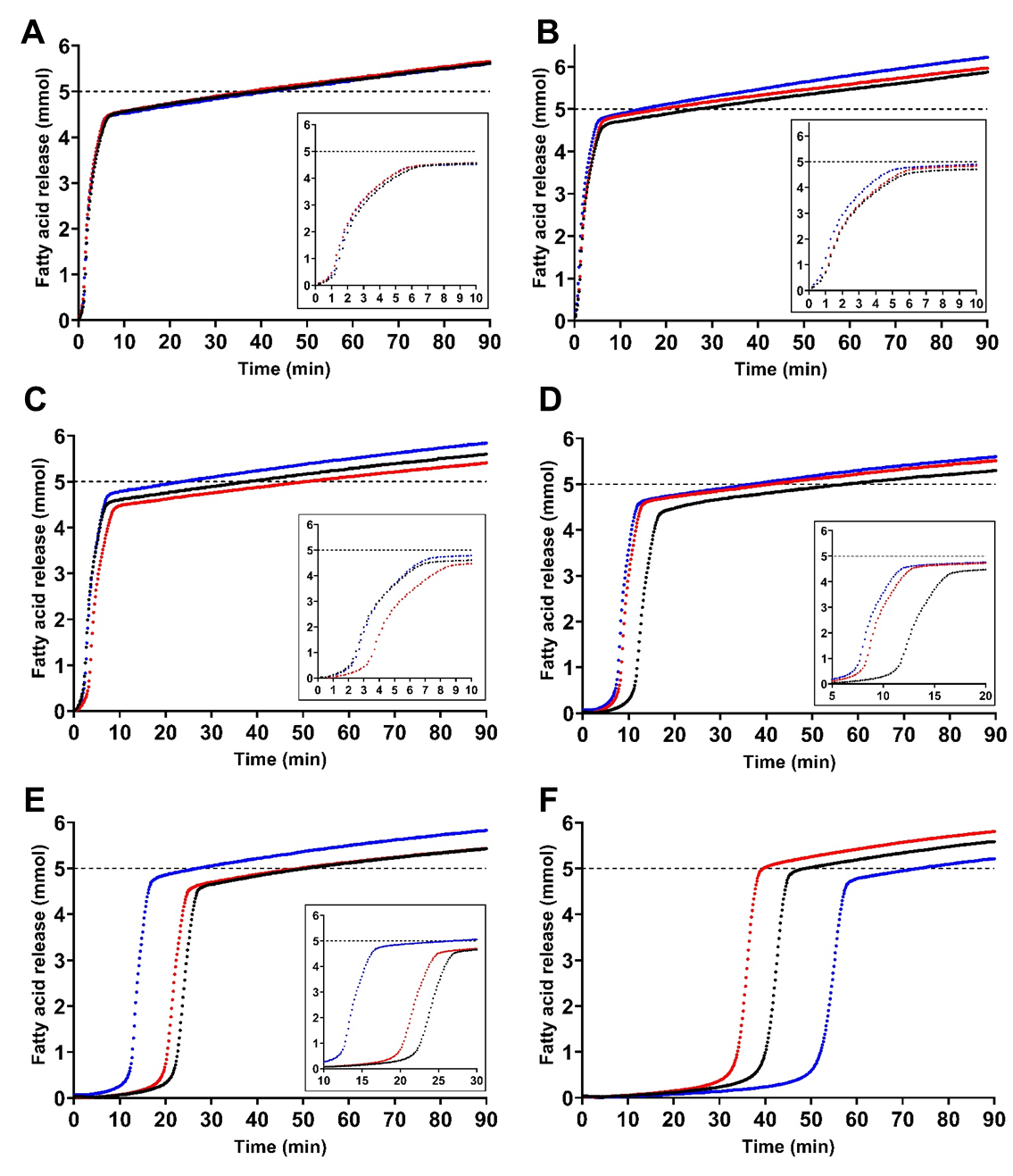


**Fig. S6** Lipolysis curves from the in vitro digestion of tricaprylin nanoemulsions with various Polysorbate 80 concentrations. A) 0.125% Polysorbate 80, B) 0.25% Polysorbate 80, C) 0.5% Polysorbate 80, D) 1% Polysorbate 80, E) 1.5% Polysorbate 80 and F) 2% Polysorbate 80. The black, red, and blue circles represent replicate 1, 2 and 3, respectively. The dotted line represents the theoretical maximum fatty acid release (i.e., 5 mmol). The lipolysis curves are corrected for the digestion of phospholipids contained in the lipolysis medium and for the fraction of unionized fatty acids as determined by ‘back titration’.


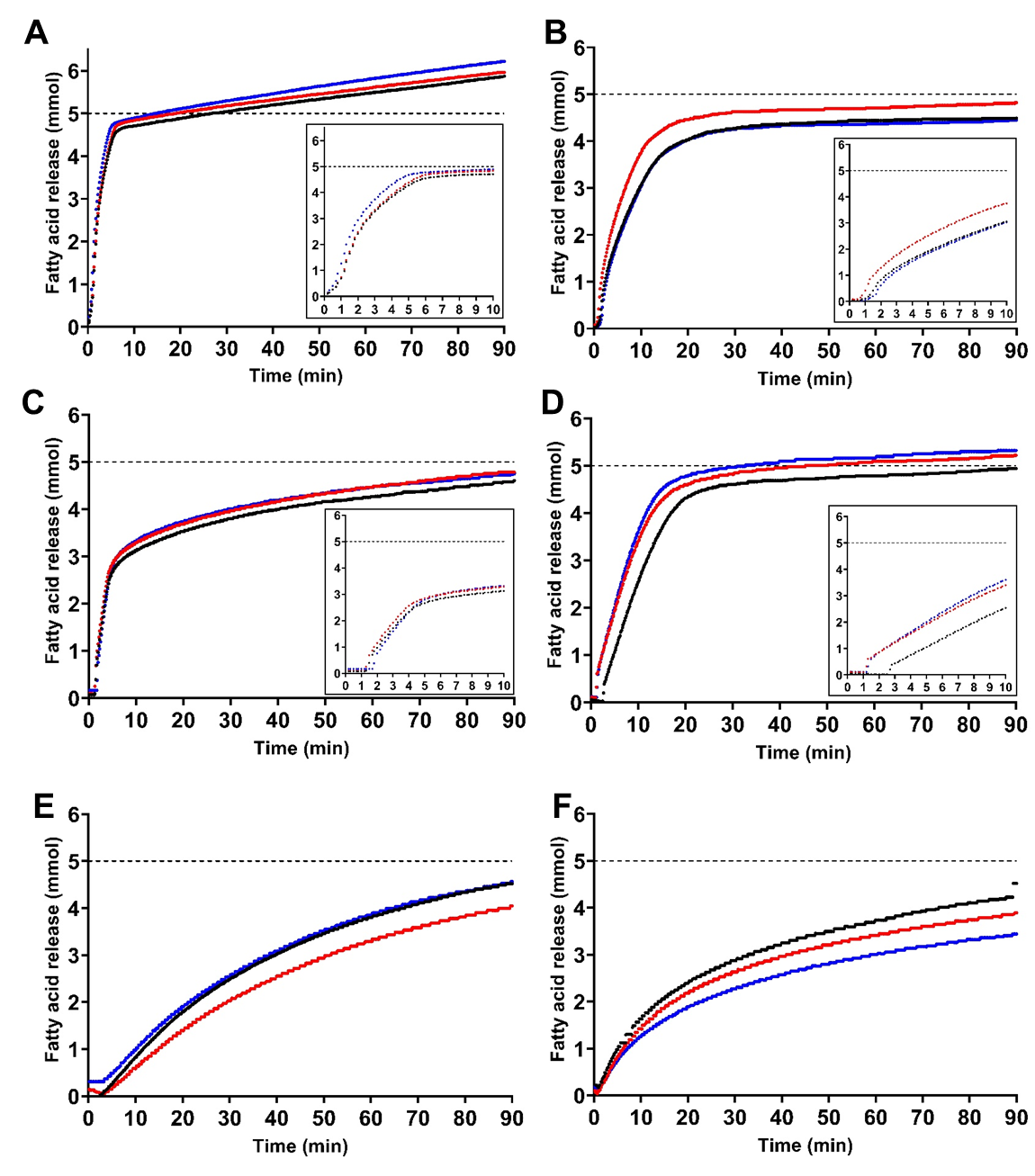


**Fig. S7** Lipolysis curves from the in vitro digestion of nanoemulsions containing one pure acylglycerol at a constant Polysorbate 80 content (0.25%). A) tricaprylin (C8), B) tricaprin (C10), C) 1,3‑dilaurin (C12), D) trilaurin (C12), E) triolein (C18:1) and F) trilinolein (C18:2). The black, red, and blue circles represent replicate 1, 2 and 3, respectively. The dotted line represents the theoretical maximum fatty acid release (i.e., 5 mmol). The lipolysis curves are corrected for the digestion of phospholipids contained in the lipolysis medium and for the fraction of unionized fatty acids as determined by ‘back titration’.


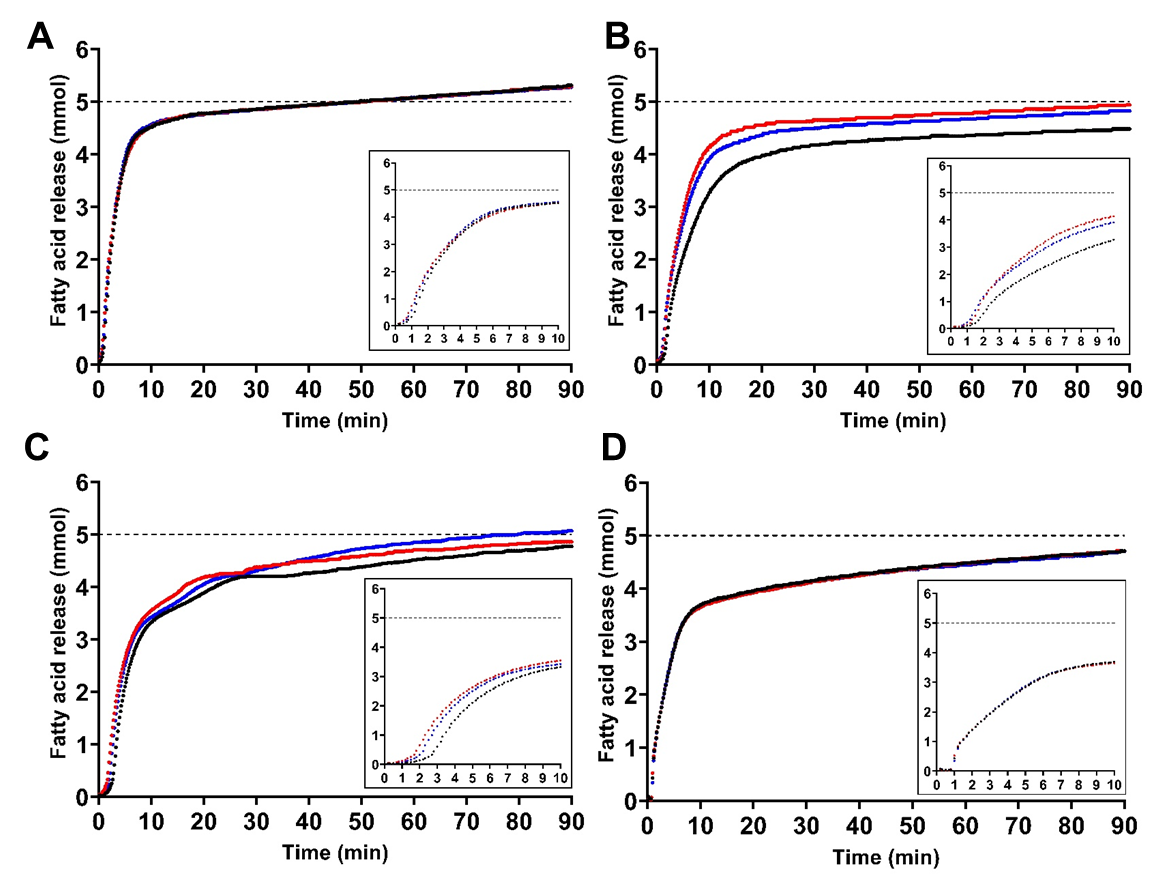


**Fig. S8** Lipolysis curves from the in vitro digestion of binary acylglycerol nanoemulsions at a constant Polysorbate 80 content (0.25%). A) 62.5 mM tricaprylin (C8) + 62.5 mM tricaprin (C10),
B) 31.25 mM tricaprylin (C8 + 93.75 mM tricaprin (C10), C) 62.5 mM tricaprylin (C8) + 62.5 mM triolein (C18:1) and D) 62.5 mM 1,3‑dilaurin (C12) + 62.5 mM trilaurin (C12). The black, red, and blue circles represent replicate 1, 2 and 3, respectively. The dotted line represents the theoretical maximum fatty acid release (i.e., 5 mmol). The lipolysis curves are corrected for the digestion of phospholipids contained in the lipolysis medium and for the fraction of unionized fatty acids as determined by ‘back titration’.


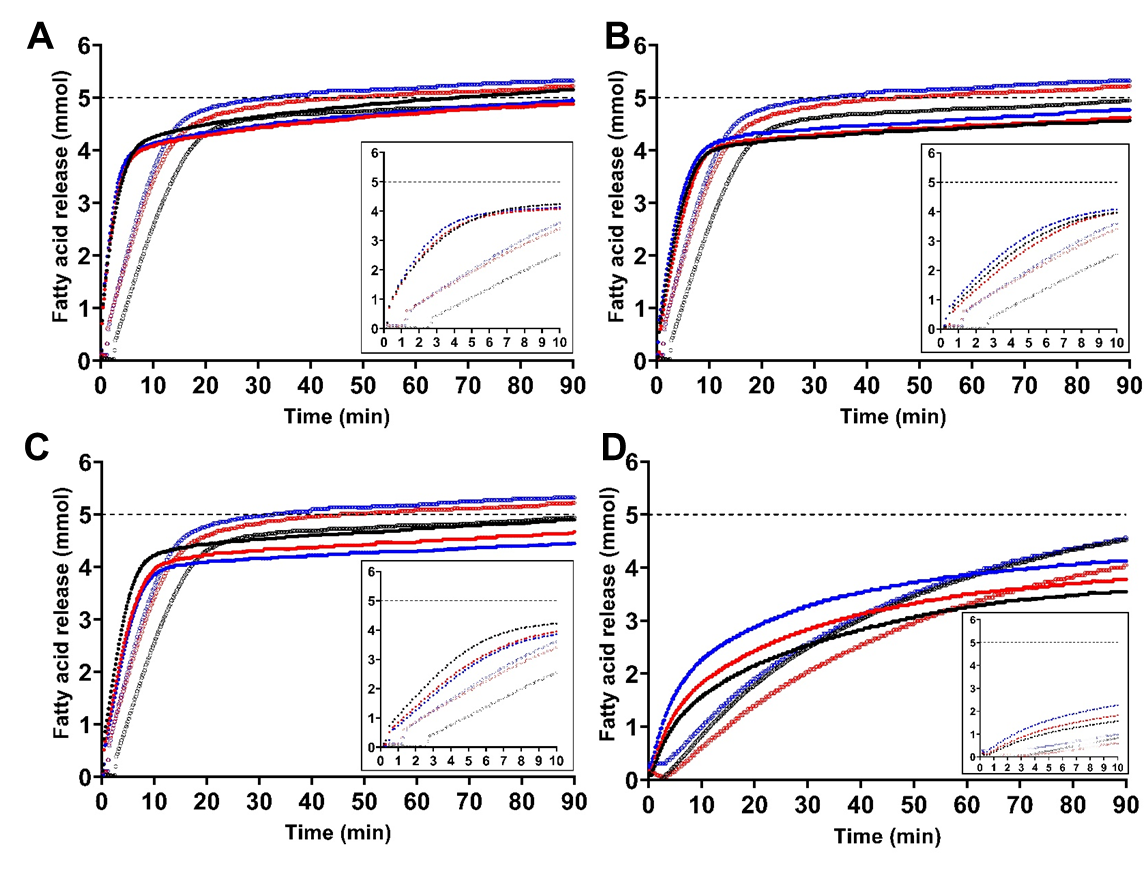


**Fig. S9** Lipolysis curves from the in vitro digestion of binary acylglycerol nanoemulsions containing small amounts of 1-monoacylglycerols at a constant Polysorbate 80 content (0.25%).
A) 25 mM 1-monocaprylin (C8) + 112.5 mM trilaurin (C12) B) 12.5 mM 1-monocaprin (C10) + 118.75 mM trilaurin (C12), C) 12.5 mM 1-monolaurin (C12) + 118.75 mM trilaurin (C12) and D) 12.5 mM 1-monocaprin (C10) + 118.75 mM triolein (C18:1). The black, red, and blue filled circles represent replicate 1, 2 and 3, respectively. For comparison, the lipolysis curves of trilaurin are given in A), B), and C) and the lipolysis curves of triolein are given in D) indicated as black, red, and blue open circles representing trilaurin/triolein replicate 1, 2, and 3. The dotted line represents the theoretical maximum fatty acid release (i.e., 5 mmol). The lipolysis curves are corrected for the digestion of phospholipids contained in the lipolysis medium and for the fraction of unionized fatty acids as determined by ‘back titration’.

**Table S6** Intrinsic lipolysis rate of acylglycerols and the degree of fatty acid ionization at the end of the in vitro lipolysis experiment.

| **Acylglycerol(s)** | **ILR (µmol/min/cm^2^)** | **Ionized fatty acids^a^ (%)** |
| --- | --- | --- |
| 1-monocaprylin | 0.142^b^ | N/A |
| Tricaprylin | 0.0063 ± 0.0003 | 78 ± 1.0 |
| 1-monocaprin | 0.0098^b^ | N/A |
| Tricaprin | 0.0028 ± 0.0004 | 42 ± 0.7 |
| 1-monolaurin | 0.0081^b^ | N/A |
| 1,3-Dilaurin (C12:0) | 0.0029 ± 0.0003 | 39 ± 0.3 |
| Trilaurin (C12:0) | 0.0009 ± 0.00009 | 38 ± 1.3 |
| Triolein (C18:1) | 0.00026 ± 0.00003 | 18 ± 1.0 |
| Trilinolein (C18:2) | 0.00044 ± 0.00004 | 14 ± 0.5 |

^a^ Determined after 90 min of lipolysis via ‘back titration’.
^b^ Predicted from the mixed intrinsic lipolysis rate of a mixture containing small amounts of 1-monoacylglycerol and trilaurin as a main component.
